# Comparative and phylogenetic analyses of the chloroplast genomes of species of Paeoniaceae

**DOI:** 10.1038/s41598-021-94137-0

**Published:** 2021-07-19

**Authors:** Liwei Wu, Liping Nie, Qing Wang, Zhichao Xu, Yu Wang, Chunnian He, Jingyuan Song, Hui Yao

**Affiliations:** 1 Key Laboratory of Bioactive Substances and Resources Utilization of Chinese Herbal Medicine, Ministry of Education, Institute of Medicinal Plant Development, Chinese Academy of Medical Sciences and Peking Union Medical College, Beijing, 100193 China; 2 Engineering Research Center of Chinese Medicine Resources, Ministry of Education, Beijing, 100193 China

**Keywords:** Molecular biology, Plant sciences

## Abstract

Plants belonging to family Paeoniaceae are not only economically important ornamental plants but also medicinal plants used as an important source of traditional Chinese medicine. Owing to the complex network evolution and polyploidy evolution of this family, its systematics and taxonomy are controversial and require a detailed investigation. In this study, three complete chloroplast genomes of sect. *Paeonia*, one of the sections of *Paeonia*, were sequenced and then analysed together with 16 other published chloroplast genomes of Paeoniaceae species. The total lengths of the chloroplast genomes of these species were 152,153–154,405 bp. A total of 82–87 protein-coding genes, 31–40 tRNA genes and 8 rRNA genes were annotated. Bioinformatics analysis revealed 61–74 simple sequence repeats (SSRs) in the chloroplast genomes, most of which have A/T base preference. Codon usage analysis showed that A/U-ending codons were more positive than C/G-ending codons, and a slight bias in codon usage was observed in these species. A comparative analysis of these 19 species of Paeoniaceae was then conducted. Fourteen highly variable regions were selected for species relationship study. Phylogenetic analysis revealed that the species of sect. *Paeonia* gathered in one branch and then divided into different small branches. *P. lactiflora*, *P. anomala*, *P. anomala* subsp. *veitchii* and *P. mairei* clustered together. *P. intermedia* was related to *P. obovata* and *P. obovata* subsp. *willmottiae*. *P. emodi* was the sister to all other species in the sect. *Paeonia*.

## Introduction

*Paeonia* is a single genus in family Paeoniaceae, which is derived from family Ranunculaceae. Since the Swedish taxonomist Carl von Linne (1735) established the genus *Paeonia*, the widely used classification systems back then, such as the Engler system and the Hooker system, placed *Paeonia* in family Ranunculaceae and remained classified under this family for over 200 years^1^. At the beginning of the twentieth century, Worsdell discovered that the stamens of *Paeonia* develop centrifugally unlike the other genera in Ranunculaceae. Consequently, *Paeonia* was finally separated from this family and classified under Paeoniaceae, but it was still placed in Order Ranunculales^2^. Numerous extensive studies on *Paeonia* covering plant morphology, anatomy, palynology, embryology, phytochemistry, chromosome number determination and karyotyping, plant reproductive genetics, and phytogeography have led to the unanimous opinion supporting the establishment of Paeoniaceae^3^. In 1946, Stern divided *Paeonia* into three sections, namely, sect. *Moutan*, sect. *Paeonia* and sect. *Onaepia*^4^. Species of sect. *Paeonia* and sect. *Onaepia* are herbaceous, whereas species of sect. *Moutan* are subshrubs^5^. Sect. *Onaepia* has only two species, which are distributed in western North America. Sect. *Paeonia*, which is the most diverse section, has over 20 species that are widely distributed in temperate climate areas of Eurasia. Seven species and two subspecies of sect. *Paeonia* are distributed in China. Eight species and six subspecies of sect. *Moutan*, which are mainly distributed in the southwest and northwest of China, are endemic to the country^6^. Paeoniaceae plants are economically important ornamental plants known for their attractive flowers. Moreover, these plants have high medicinal value. Monoterpene glycosides, flavonoids, tannins, stilbene, triterpenoids and other compounds have been found in species of Paeoniaceae^7–16^. These compounds have antioxidation, antitumor and antipathogenic properties and play a role in immune system regulation, cardiovascular system protection, central nervous system protection and optic nerve protection^17–23^.

Section *Paeonia* is the only section with chromosome ploidy changes. Species of sect. *Moutan* and sect. *Onaepia* are all diploid, whereas species of sect. *Paeonia* have diploid and tetraploid chromosomes^24^. The existence of different ploidy in sect. *Paeonia* makes its phylogenetic relationship very complicated. The tetraploid species in this section were initially thought to be homologous tetraploids^4,25^. However, morphological, cytogenetic and molecular phylogenetic studies showed that this section has allotetraploid species, and some tetraploid groups originated from interspecific hybridisation of two different subgroups^26–35^. Although evidence exists that tetraploid groups are mostly of heterogenetic origin, the origin and classification of sect. *Paeonia* are controversial because of the consistent karyotype, similar morphology and overlapping geographical distributions of species belonging to this section^36^. In classical taxonomy, the use of phenotypic traits alone to infer phylogenetic relationships between taxa with different genotypes is replete with problems^33^. These taxonomic problems caused by different understandings of morphological variations can be resolved using molecular markers independent of morphological features. Early researchers focused on the study of DNA fragment-labelling techniques or phylogenetic analysis based on nuclear or chloroplast DNA fragments^37–43^. Based on the results of ITS and *matK* phylogenetic analysis, Sang et al.^33^ constructed the reticular evolution model diagram of sect. *Paeonia*. However, phylogenetic analysis showed that the results of ITS and *matK* only provided partial information on the origin of allotetraploid groups^36^. Owing to chromosomal ploidy, complex network evolution and polyploidy evolution^26^, limited nuclear or chloroplast DNA fragments cannot provide sufficient phylogenetic information to effectively solve the interspecies relationships of sect. *Paeonia*. Research on the genetic diversity of sect. *Paeonia* is relatively slow, and related studies at the molecular level are not comprehensive^44^. The relationships among species of sect. *Moutan* have such problems because of their complex evolution and phylogeny^32,33,45–49^.

The chloroplast genome is an organ independent of the nuclear genome. The chloroplast genome can be maternally inherited, has highly conserved gene content and order and has a slow molecular evolution and a low recombination rate, making it an ideal material for species authentication and phylogenetic studies^50–53^. Most of the chloroplast genomes of angiosperms have a circular tetrad structure, which consists of two inverted repeats (IRs), a large single copy (LSC) and a small single copy (SSC)^54^. The chloroplast genome has been applied to phylogenetic analysis and species identification of multiple plants^55–60^. Therefore, we can use the chloroplast genome to analyse the relationship among species of Paeoniaceae. In this study, the complete chloroplast genomes of three species of sect. *Paeonia* were sequenced and analysed together with other Paeoniaceae species. Comparative and phylogenetic analyses were then performed on the chloroplast genomes of 19 species of Paeoniaceae, including 8, 10 and 1 chloroplast genomes of sect. *Paeonia*, sect. *Moutan* and sect. *Onaepia*, respectively.

## Results and discussion

### Statistics and genetic composition of 19 Paeoniaceae chloroplast genomes

**Figure 1:**
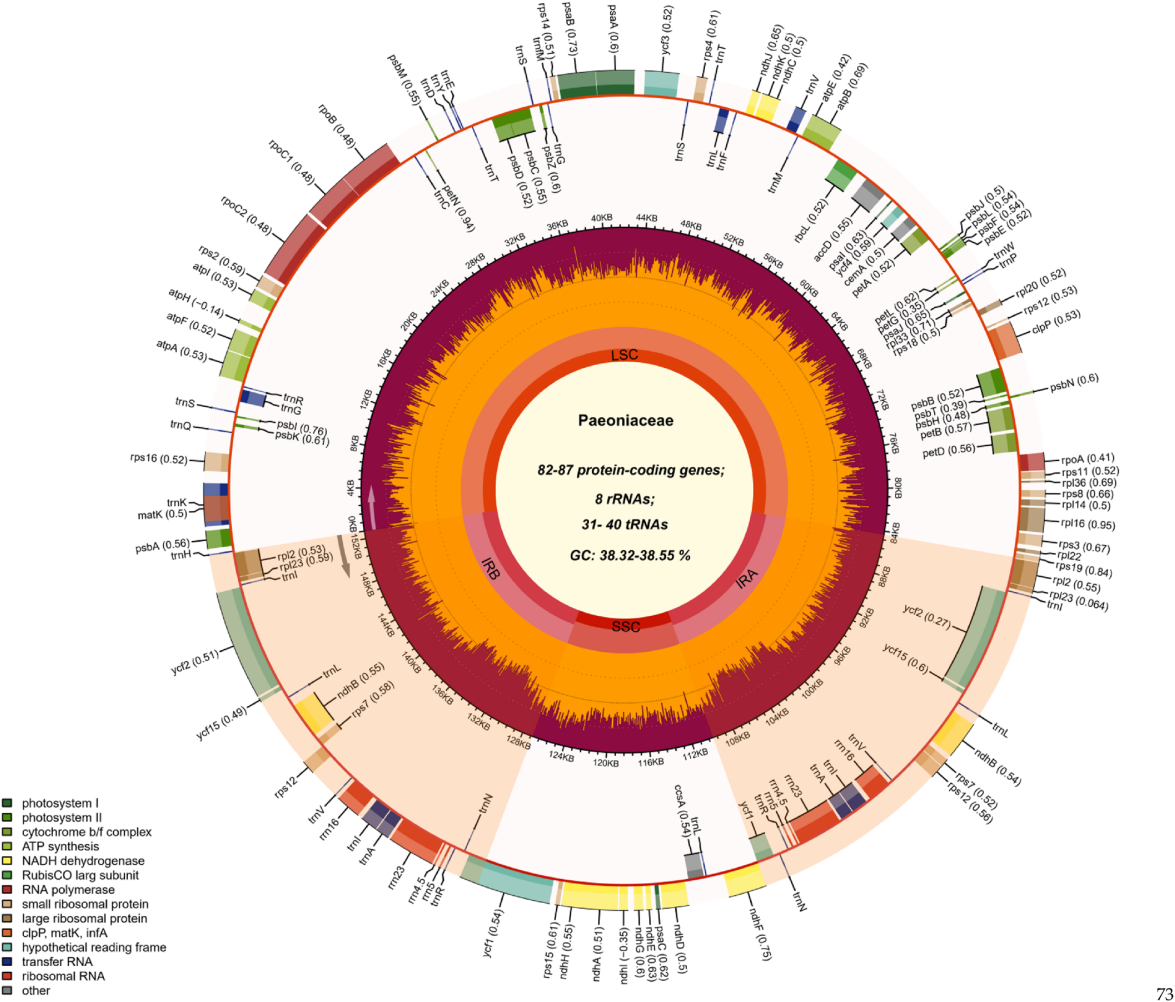
Chloroplast genome map of Paeoniaceae species, using *P. intermedia* as the template. The gradient GC content of the genome was plotted in the second circle with zero level based on the outer circle. The gene names and their codon usage bias were labeled on the outermost layer. The gene specific GC content was depicted with the proportion of shaded areas. Represented with arrows, the transcription directions for the inner and outer genes were listed clockwise and anticlockwise, respectively.

The chloroplast genomes of 19 Paeoniaceae species were all classical tetrad structures containing an LSC, an SSC and a pair of IRs (Fig. 1). The total lengths of the chloroplast genomes were 152,153 (*P. ostii*)–154,405 bp (*P. delavayi*). Total GC contents ranged from 38.32% (*P. ostia* and *P. rockii*) to 38.55% (*P. brownii*). The lengths of the IR, LSC and SSC regions were 24,729–26,049, 84,241–86,316, and 16,679–17,059 bp, respectively. The GC contents of the four regions were not balanced. The IR regions had the highest GC content (42.98–43.16%), followed by the LSC (36.63–36.83%) and the SSC regions (32.57–33.02%) (Supplementary Table S1).

**Figure 2:**
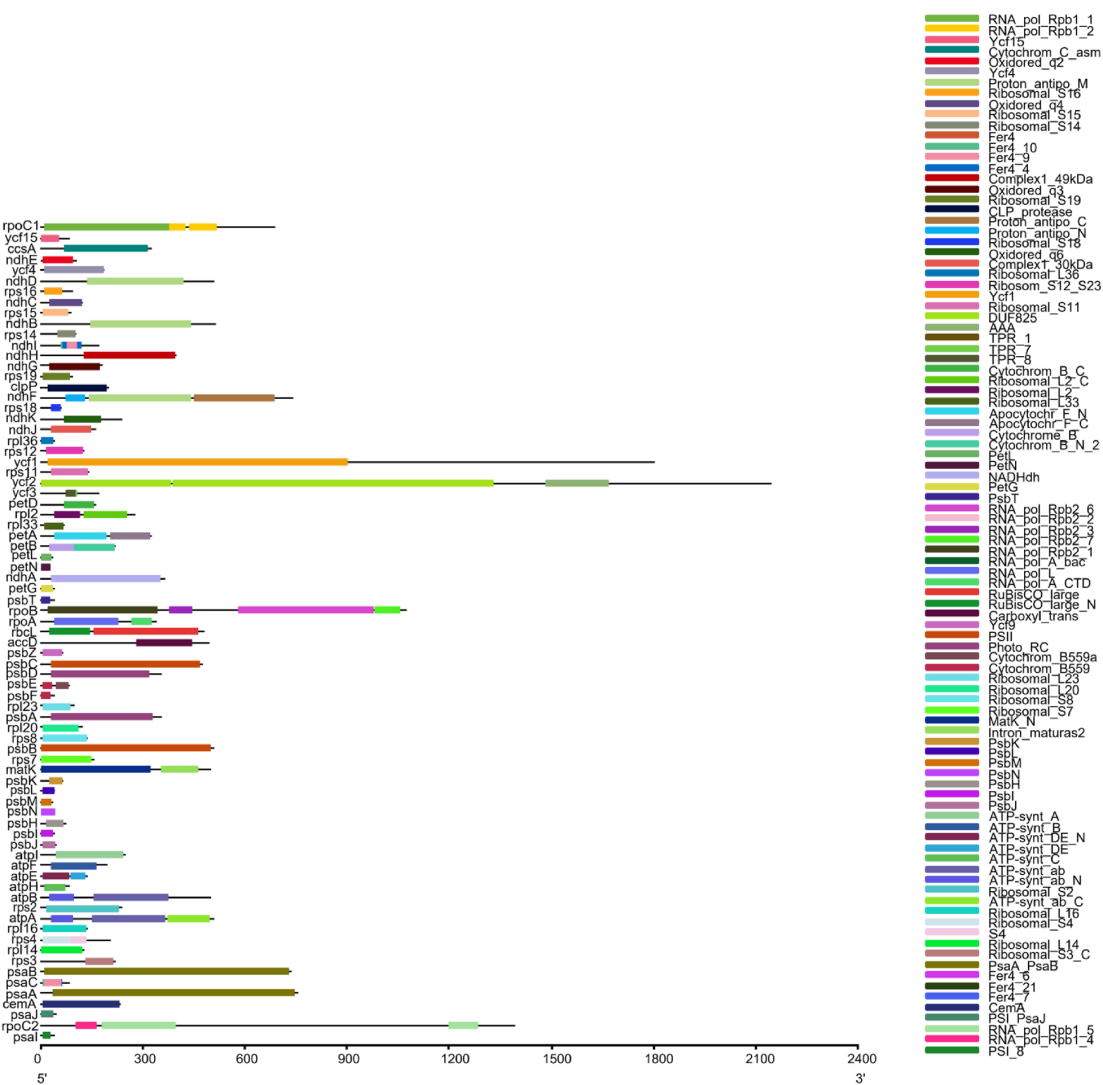
Protein functional domains of protein-coding genes in Paeoniaceae species.

The structure and gene composition of the chloroplast genomes of Paeoniaceae species can be divided into 14 categories (Fig. 1). The proteins produced by different combinations of domains are different in nature, and the identification of protein domains is particularly important for analysing protein functions. The protein functional domains of protein-coding genes in the Paeoniaceae species are listed in Fig. 2. A total of 82–87 protein-coding genes, 31–40 tRNA genes and 8 rRNA genes were annotated in the Paeoniaceae species. In the three chloroplast genomes obtained in this study, seven protein-coding genes (*rpl2*, *rpl23*, *ycf2*, *ycf15*, *ndhB*, *rps7* and *rps12*), seven tRNAs (*trnI-CAU*, *trnL-CAA*, *trnV-GAC*, *trnI-GAU*, *trnA-UGC*, *trnR-ACG* and *trnN-GUU*) and four rRNAs (*rrn16*, *rrn23*, *rrn4.5* and *rrn5*) were located in the IR regions. Introns play an important role in the regulation of gene expression, and it can enhance the expression of exogenous genes at specific loci of plants and produce ideal agronomic traits^61^. Among the protein-coding genes, 18 genes contained introns, of which 3 genes (*clpP*, *rps12* and *ycf3*) contained two introns, whereas the remaining 15 genes contained only one intron (Supplementary Table S2). *rps12* gene is a trans-splicing gene with a 5′ end in the LSC region and a 3′ end in the IR region, similar to that of many other plants^62–64^.

### Analysis of codon usage bias of the chloroplast genomes

The choice of synonymous codons for amino acids encoded by an organism’s genes is not completely random, and there is codon usage bias^65^. Codon usage bias not only plays an important regulatory role in gene expression level but also helps to improve the accuracy and efficiency of translation^66,67^. In addition to being affected by selection and mutation, codon usage is affected by tRNA abundance, base composition, gene position on chromosomes, gene length and expression level, amino acid hydrophobicity and mRNA secondary structure^68–74^. Paeoniaceae species can be divided into groups A and B according to the codon usage of chloroplast genomes. Group B included *P. lactiflora*, *P. obovata*, *P. rockii* and *P. rockii* subsp. *taibaishanica*, whereas group A included the remaining 15 species.

**Figure 3:**
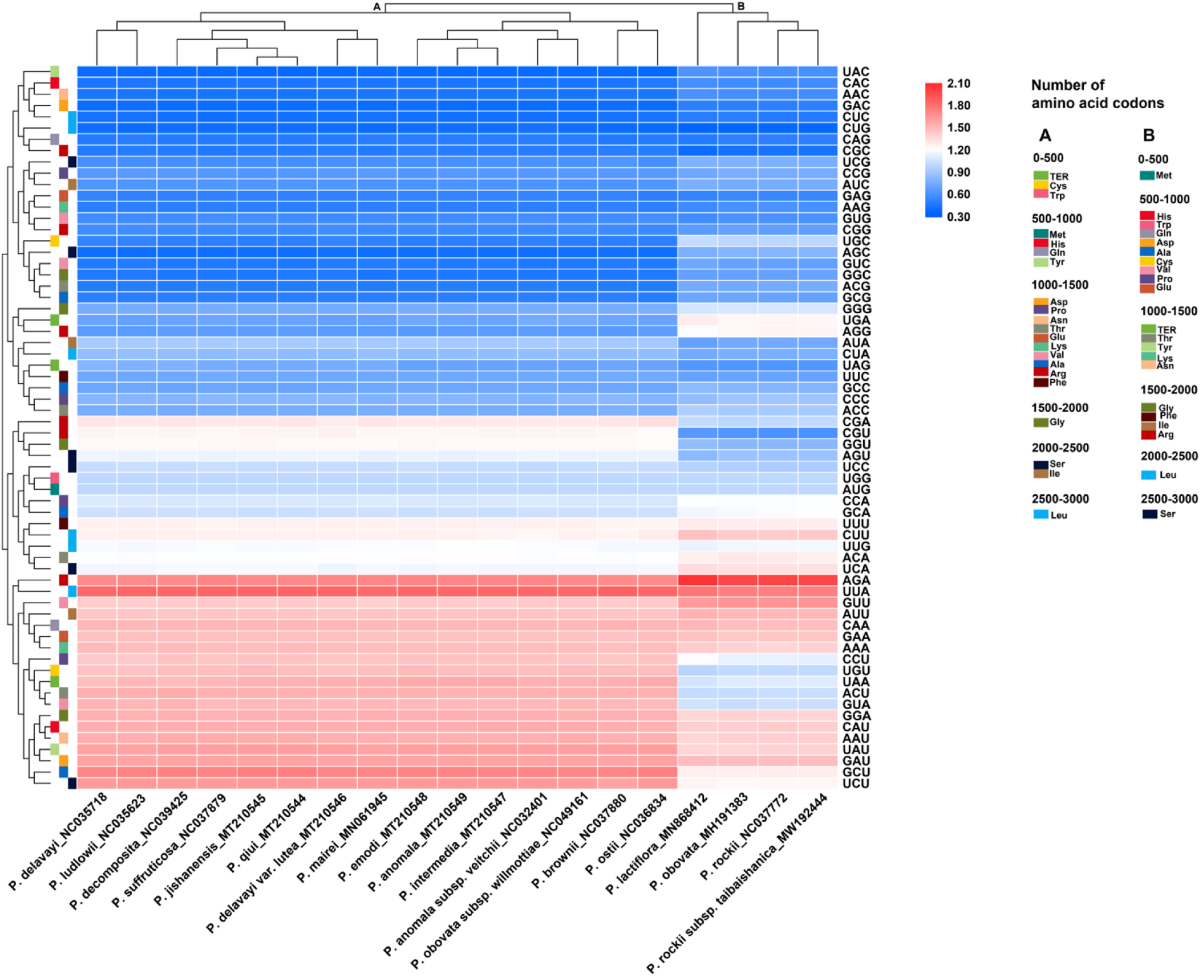
Heat map of relative synonymous codon usage (RSCU) values among Paeoniaceae species.

The relative synonymous codon usage (RSCU) of the chloroplast genomes of Paeoniaceae species was calculated on the basis of all protein-coding genes (Supplementary Table S3). Results showed that the chloroplast genomes of Paeoniaceae species contained 64 types of codons encoding 20 amino acids. In group A, of all amino acid codons, leucine had the highest number of codons, whereas cysteine had the lowest number of codons. Thirty-one codons were found with an RSCU of > 1, of which 29 were A/U-ending codons; 33 codons were found with an RSCU of ≤ 1, of which 30 were G/C-ending codons. The highest RSCU value was recorded for UUA and the lowest for UAC, which encode leucine and tyrosine, respectively. In group B, serine had the highest number of codons, and methionine had the lowest number of codons. Furthermore, 29 (*P. obovata*) and 30 (*P. lactiflora*, *P. rockii* and *P. rockii* subsp. *taibaishanica*) codons were found with an RSCU of > 1, of which 26 (*P. lactiflora* and *P. obovata*) and 27 (*P. rockii* and *P. rockii* subsp. *taibaishanica*) were A/U-ending codons. Moreover, 34 (*P. lactiflora*, *P. rockii* and *P. rockii* subsp. *taibaishanica*) and 35 (*P. obovata*) codons were found with an RSCU of ≤ 1, of which 29 (*P. obovata*) and 32 (*P. lactiflora*, *P. rockii* and *P. rockii* subsp. *taibaishanica*) were G/C-ending codons. The highest RSCU value was recorded for AGA and the lowest for CUG, which encode arginine and leucine, respectively. To conclude, A/U-ending codons were more positive than G/C-ending codons (Fig. 3).

GC refers to the total content of all codons G and C, and GC3s pertains to the frequency of G and C bases in the third codon base of synonymous codons encoding the same amino acid. GC reflects the strength of directional mutation pressure, and GC3s is closely related to codon bias^75^. The main difference in synonymous codons is reflected in the third base, and a change in this base of codons usually does not cause changes in encoded amino acids. Therefore, the selection pressure on the third base of the codon is less selective. GC3s is used as an important basis for analysing codon usage pattern^76^. GC and GC3s in the codons of these 19 chloroplast genomes were all less than 0.5, indicating that the chloroplast genomes of Paeoniaceae species tended to use A/T bases and A/T-ending codons. Codon adaptation index values and effective number of codon values indicated a slight bias in codon usage in the Paeoniaceae species. Frequency of optimal codons was relatively low. In addition, the hydrophobicity of the protein (i.e., Gravy) and the aromatic protein (i.e., Aromo) had little effect on codon usage bias. Compared with those in group A, the species in group B had higher GC and GC3s contents and slighter codon usage bias, and Gravy and Aromo had a greater influence on codon usage bias (Table 1).

**Table 1:** Codon usage of the Paeoniaceae species.

Species	T3s	C3s	A3s	G3s	GC3s	GC	CAI	ENc	Fop	Gravy	Aromo	L_sym	L_aa
*P. intermedia*	0.4582	0.1788	0.4213	0.1914	0.283	0.386	0.166	50.92	0.354	− 0.089477	0.111029	24,652	25,714
*P. emodi*	0.4585	0.1795	0.4207	0.1909	0.284	0.387	0.166	50.91	0.354	− 0.088465	0.111016	24,558	25,618
*P. anomala*	0.4581	0.1791	0.4214	0.191	0.283	0.386	0.166	50.9	0.354	− 0.089123	0.110835	24,651	25,714
*P. anomala* subsp*. veitchii*	0.4583	0.1783	0.4216	0.1915	0.283	0.386	0.166	50.9	0.354	− 0.088383	0.110857	24,698	25,763
*P. mairei*	0.4574	0.1796	0.421	0.1907	0.284	0.387	0.166	50.96	0.354	− 0.083818	0.110868	24,936	26,004
*P. obovata* subsp. *willmottiae*	0.4583	0.179	0.4206	0.1916	0.284	0.387	0.166	50.95	0.354	− 0.088775	0.111241	24,655	25,719
*P. jishanensis*	0.4595	0.1782	0.4223	0.1897	0.282	0.386	0.166	50.8	0.354	− 0.08939	0.111236	24,645	25,711
*P. decomposita*	0.4593	0.1784	0.4219	0.1895	0.282	0.386	0.166	50.81	0.354	− 0.081256	0.111128	24,722	25,790
*P. qiui*	0.4596	0.1783	0.4223	0.1897	0.282	0.386	0.166	50.79	0.354	− 0.090311	0.111159	24,644	25,711
*P. ostii*	0.4596	0.18	0.4214	0.1892	0.283	0.386	0.167	50.87	0.354	− 0.086684	0.1115	24,642	25,713
*P. suffruticosa*	0.4586	0.1789	0.422	0.1905	0.283	0.386	0.166	50.86	0.354	− 0.088544	0.111268	24,493	25,551
*P. ludlowii*	0.4569	0.1807	0.4208	0.1904	0.284	0.387	0.166	51.05	0.355	− 0.084929	0.110735	24,942	26,017
*P. delavayi* var. *lutea*	0.4572	0.1805	0.4214	0.1899	0.284	0.387	0.167	50.99	0.355	− 0.089308	0.110296	24,160	25,214
*P. delavayi*	0.4571	0.1802	0.4212	0.1906	0.284	0.387	0.166	51.03	0.354	− 0.082886	0.110866	24,763	25,833
*P. brownii*	0.4566	0.1813	0.4209	0.191	0.285	0.387	0.166	51.03	0.354	− 0.089699	0.110977	24,541	25,600
*P. lactiflora*	0.4117	0.2366	0.3993	0.2224	0.351	0.395	0.16	53.75	0.363	− 0.308232	0.146809	23,536	24,440
*P. obovata*	0.413	0.2353	0.3955	0.2294	0.354	0.395	0.158	54.1	0.359	− 0.320514	0.147034	23,524	24,457
*P. rockii*	0.4139	0.2347	0.3959	0.2289	0.353	0.394	0.158	54	0.359	− 0.321144	0.147478	23,538	24,485
*P. rockii* subsp. *taibaishanica*	0.4138	0.2348	0.3962	0.2283	0.352	0.394	0.158	54.01	0.359	− 0.319911	0.147729	23,538	24,484

*T3s/C3s/A3s/G3s/GC3s* the thymine/cytosine/adenine/guanine/GC content at synonymous third codon position, *GC* the total GC content, *CAI* codon adaptation index, *ENc* effective number of codons, *Fop* Frequency of optimal codons, *Gravy* the influence of protein hydrophobicity on codon usage bias, *Aromo* the influence of aromatic protein on codon usage bias, *L_sym* number of synonymous codons, *L_aa* total number of synonymous and non-synonymous codons.

### Long repeat sequences and SSRs

**Figure 4:**
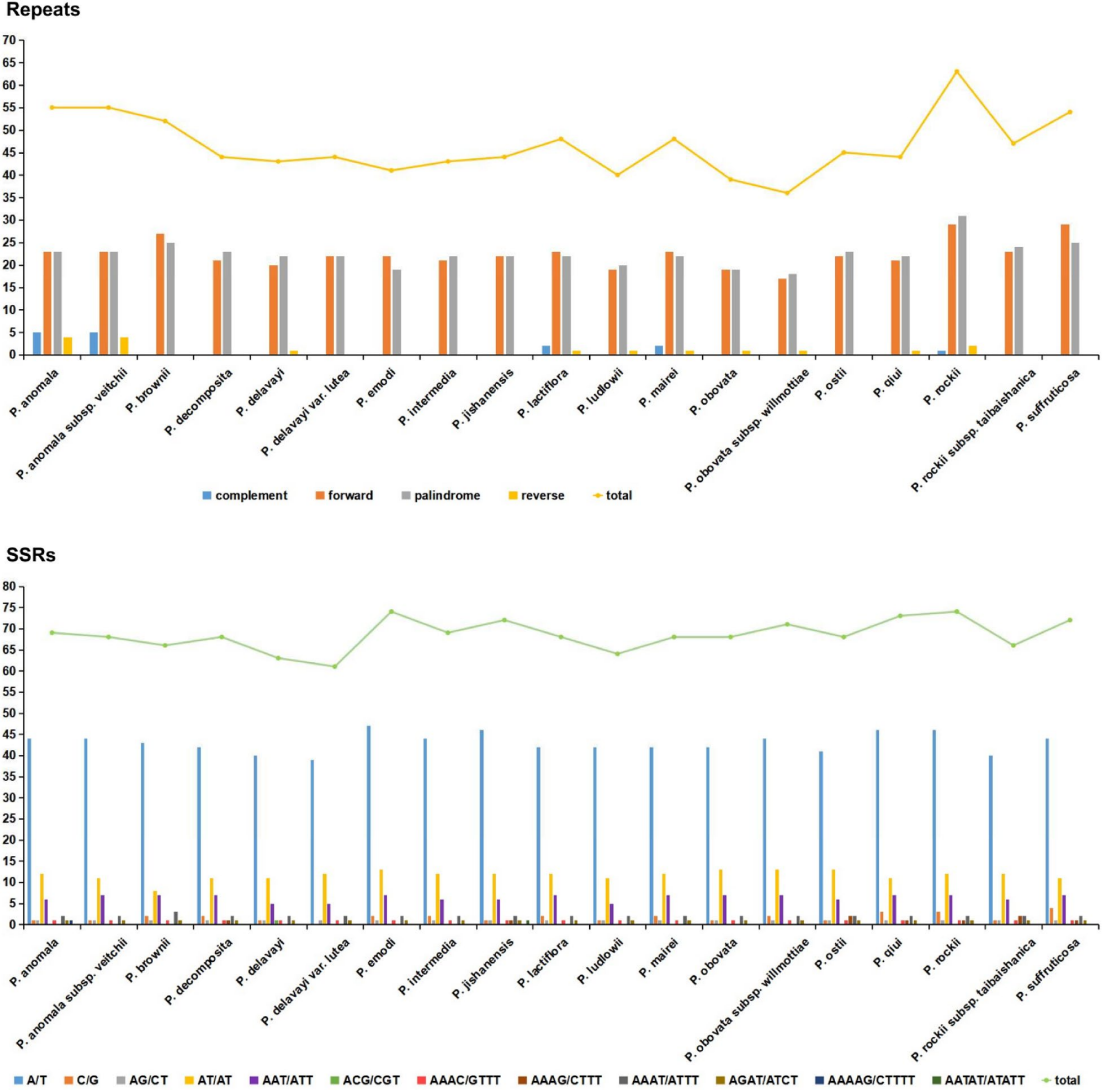
Types and amounts of repeats and SSRs in Paeoniaceae chloroplast genomes.

Long repeats play an important role in genome rearrangement and are often used to study phylogenetic relationships between species; moreover, they promote intermolecular recombination in the chloroplast genomes of plants to produce diversity^77^. Long repeat sequences include forward, palindrome, reverse and complement. For all repeat types, repeat length is ≥ 30 bp and sequence similarity is ≥ 90%. In Paeoniaceae species, our results revealed 36–63 long repeats, most of which were forward (17–29) and palindrome (18–31) repeats. Complement repeats were the least distributed and found only in *P. anomala*, *P. anomala* subsp. *veitchii*, *P. lactiflora*, *P. mairei* and *P. rockii*. In addition, the length of these repeats was mainly within the range of 30–39 bp. Repeats with a length of ≥ 70 bp only existed in *P. brownie*, *P. ostia* and *P. rockii* subsp. *taibaishanica* (Fig. 4) (Supplementary Table S4).

SSRs, also known as microsatellite sequences, are a kind of tandem repeat sequences consisting of 1–6 repeating nucleotide units that are widely distributed throughout chloroplast genomes^78^. Owing to their high polymorphism, SSRs are increasingly used as molecular markers, in species identification and in studying population genetics and phylogenetic relationships^79–81^. A total of 61–74 SSRs were identified in the chloroplast genomes of the Paeoniaceae species. In addition, the base composition of the repeating motifs from mononucleotide repeats to trinucleotide repeats had a certain base preference, mainly the repeating motifs rich in A–T. In these SSRs, mononucleotide repeats were the largest in number, which were found 39–49 times in these chloroplast genomes. A/T repeats (91.7–100%) were the most common mononucleotide repeats, whereas the majority of dinucleotide repeat sequences comprised of AT/AT repeats (88.9–92.9%), and all of trinucleotide repeats were AAT/ATT, except for *P. delavayi*. These results were consistent with A-T enrichment in complete chloroplast genomes^82^. Moreover, compared with polyC and polyG, polyA and polyT occupy a relatively high proportion in the SSRs of many plants^83^. ACG/CGT, AAAAG/CTTTT and AATAT/ATATT were found to be unique in *P. delavayi*, *P. anomala* and *P. jishanensis*, respectively (Fig. 4) (Supplementary Table S5).

### Comparative analysis of chloroplast genomes of Paeoniaceae

**Figure 5:**
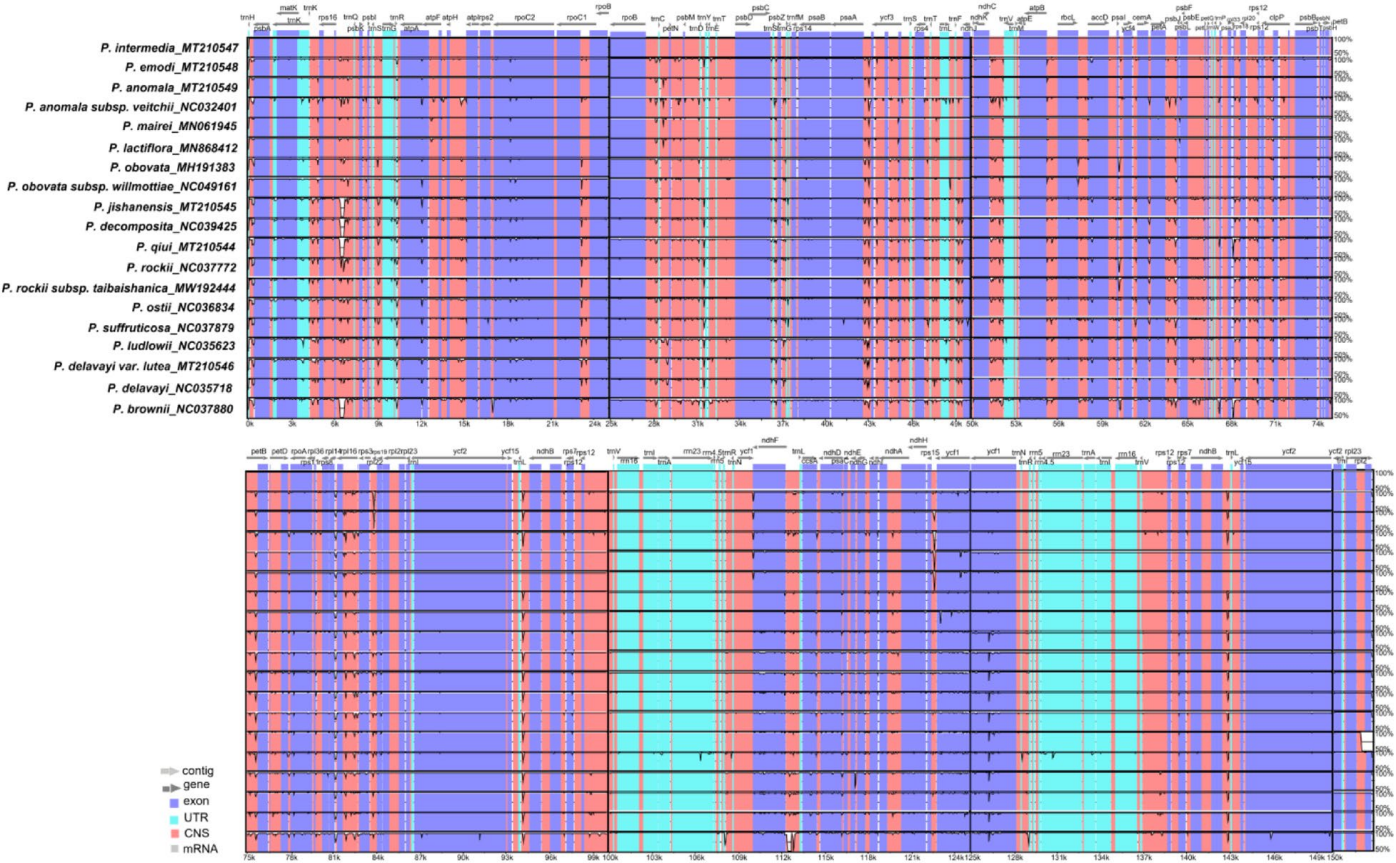
Global alignment of chloroplast genomes of 19 Paeoniaceae species. The x-axis represents the coordinates in the chloroplast genome. The y-axis indicates the average percent identity of sequence similarity in the aligned regions, ranging between 50 and 100%. Genome regions are color coded as protein coding, rRNA coding, tRNA coding, or conserved noncoding sequences (CNS).

In this study, the complete chloroplast genomes of 19 species of Paeoniaceae were compared using mVISTA^84^ with the *P. intermedia* genome as the reference genome (Fig. 5). Overall, the comparative genomic analysis revealed that the 19 Paeoniaceae chloroplast genomes were relatively conserved. Intergenic spacers and intron regions showed more variations than protein-coding regions. Most protein-coding regions had a very high degree of conservation (most had > 90% similarity), and rRNA genes (*rrn4.5*, *rrn5*, *rrn16* and *rrn23*) were highly conserved with almost no variation. Variations in the SSC and LSC regions were considerably greater than those in the IR regions, similar to studies in other plants^85–89^.

**Figure 6:**
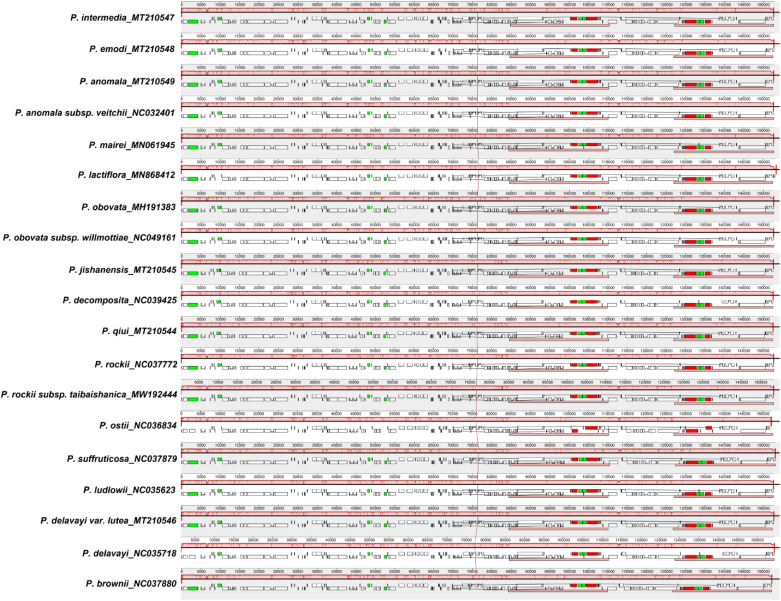
Co-linear analysis of 19 Paeoniaceae species chloroplast genomes. Local collinear blocks are represented by blocks of the same color connected by lines.

A co-linear analysis of the 19 Paeoniaceae chloroplast genomes was conducted with the *P. intermedia* genome as the reference genome. Results showed that the entire genome sequence was a homologous region with no big indels. The 19 chloroplast genomes connected with a line, indicating that the chloroplast genomes of these species were relatively conserved, and no rearrangement occurred in gene organisation (Fig. 6).

**Figure 7:**
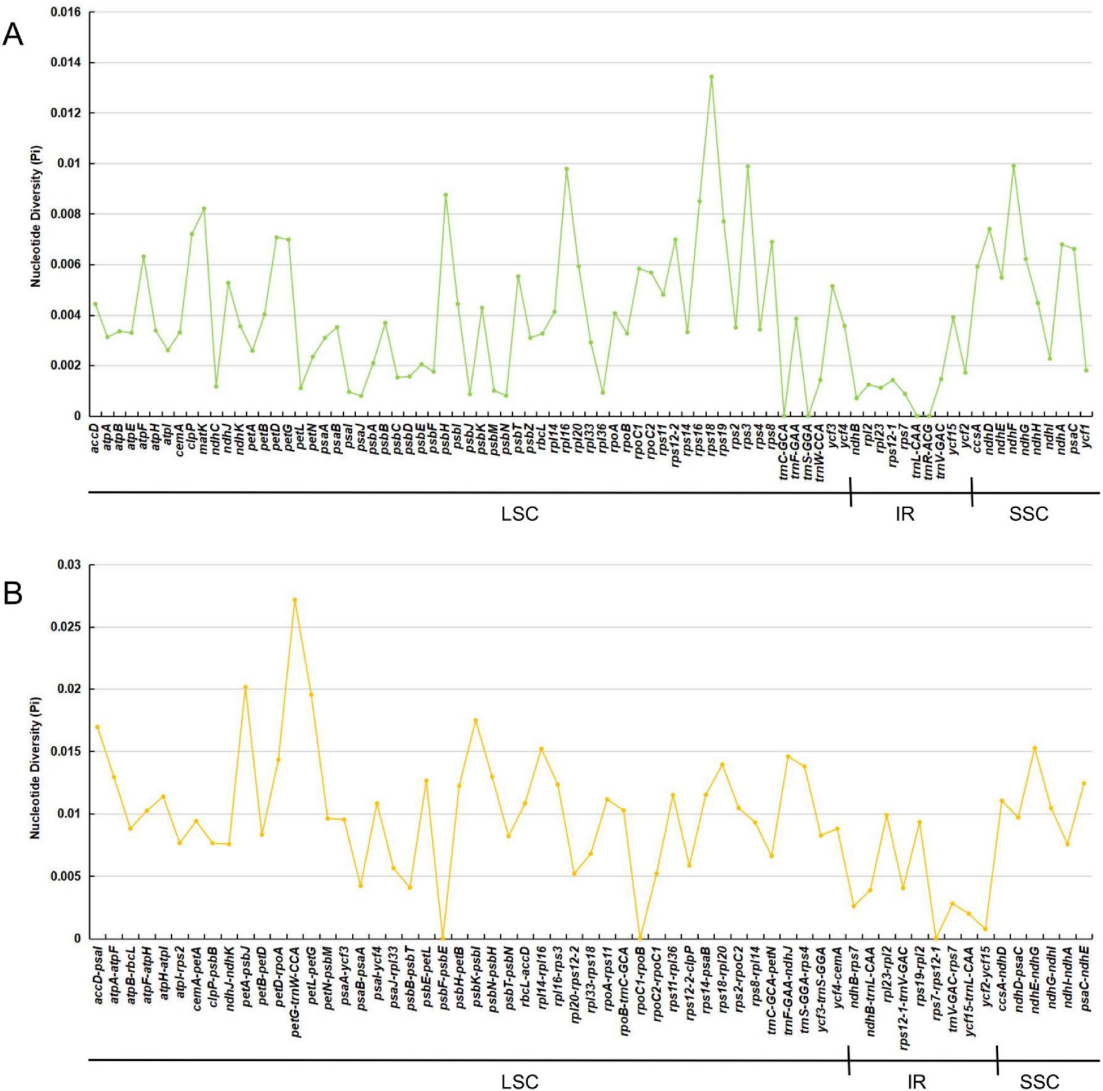
Nucleotide diversity (Pi) of shared various regions in 19 Paeoniaceae species chloroplast genomes. (**A**) Pi values in the genes regions. (**B**) Pi values in the intergenic spacers regions.

Mutational hotspots of shared genes and intergenic spacers of the chloroplast genomes of the 19 Paeoniaceae species were identified by DnaSP^90^. The intergenic spacers had more polymorphisms (average Pi = 0.00955) than the gene regions (average Pi = 0.00393). Moreover, the largest nucleic acid variation was observed in the SSC regions (average Pi in intergenic spacers = 0.01107; average Pi in gene regions = 0.00568), followed by that in the LSC regions (average Pi in intergenic spacers = 0.01045; average Pi in gene regions = 0.00408) and that in the IR regions (average Pi in intergenic spacers = 0.00391; average Pi in gene regions = 0.00124). These results were consistent with those of mVISTA analysis. Seven protein-coding genes (*rps18*, *ndhF*, *rps3*, *rpl16*, *psbH*, *rps16* and *matK*) positioned at the single copy regions exhibited high Pi values (> 0.008) (Fig. 7A). By comparison, seven intergenic spacers (*petG*-*trnW-CCA*, *petA*-*psbJ*, *petL*-*petG*, *psbK*-*psbI*, *accD*-*psaI*, *ndhE*-*ndhG* and *rpl14*-*rpl16*) showed high diversity values (> 0.015) (Fig. 7B).

### Phylogenetic analysis of Paeoniaceae

**Figure 8:**
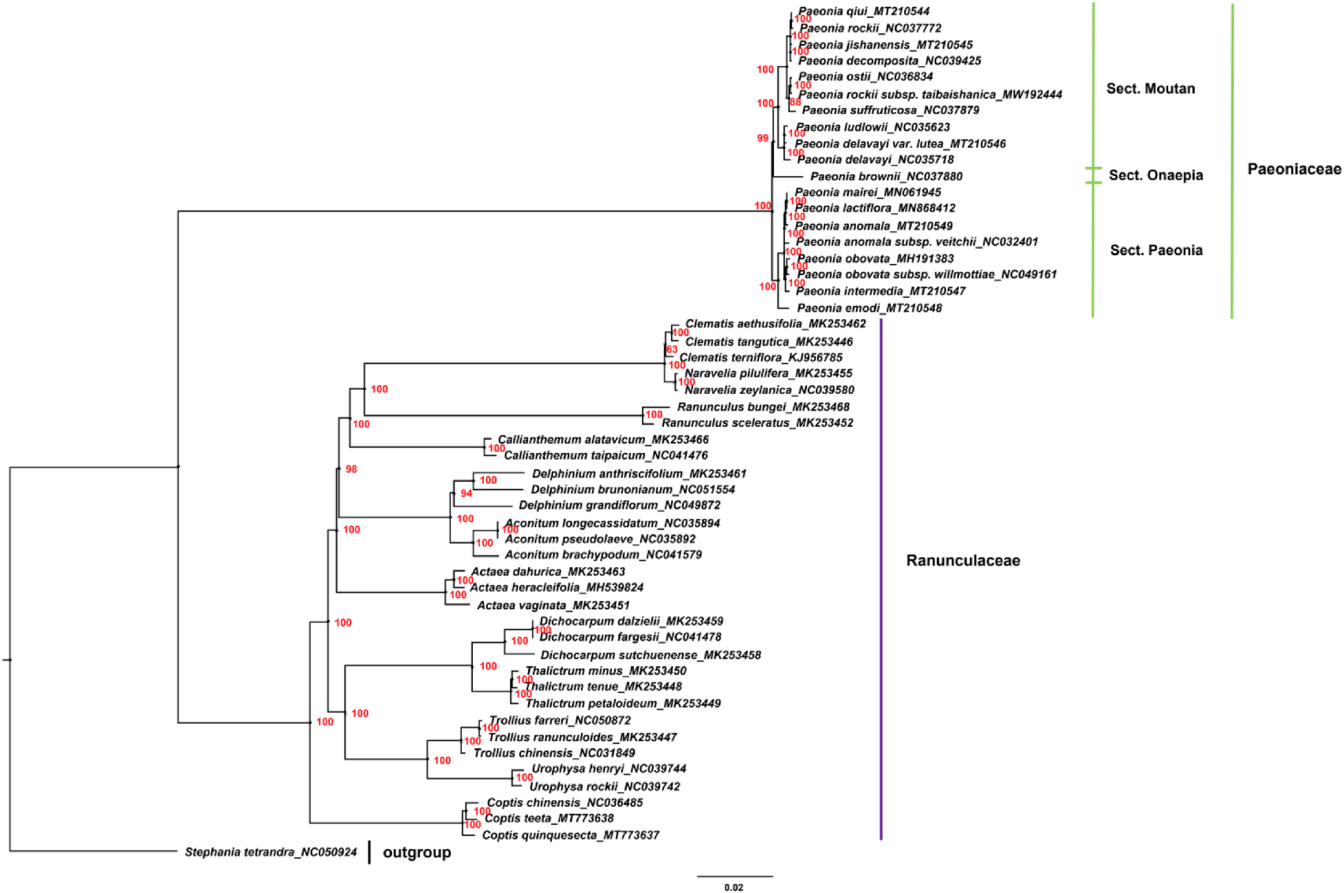
Phylogenetic tree constructed using Maximum Likelihood (ML) method based on the complete chloroplast genome sequences of 19 Paeoniaceae species and 32 Ranunculaceae species. Red numbers at nodes are values for bootstrap support.

**Figure 9:**
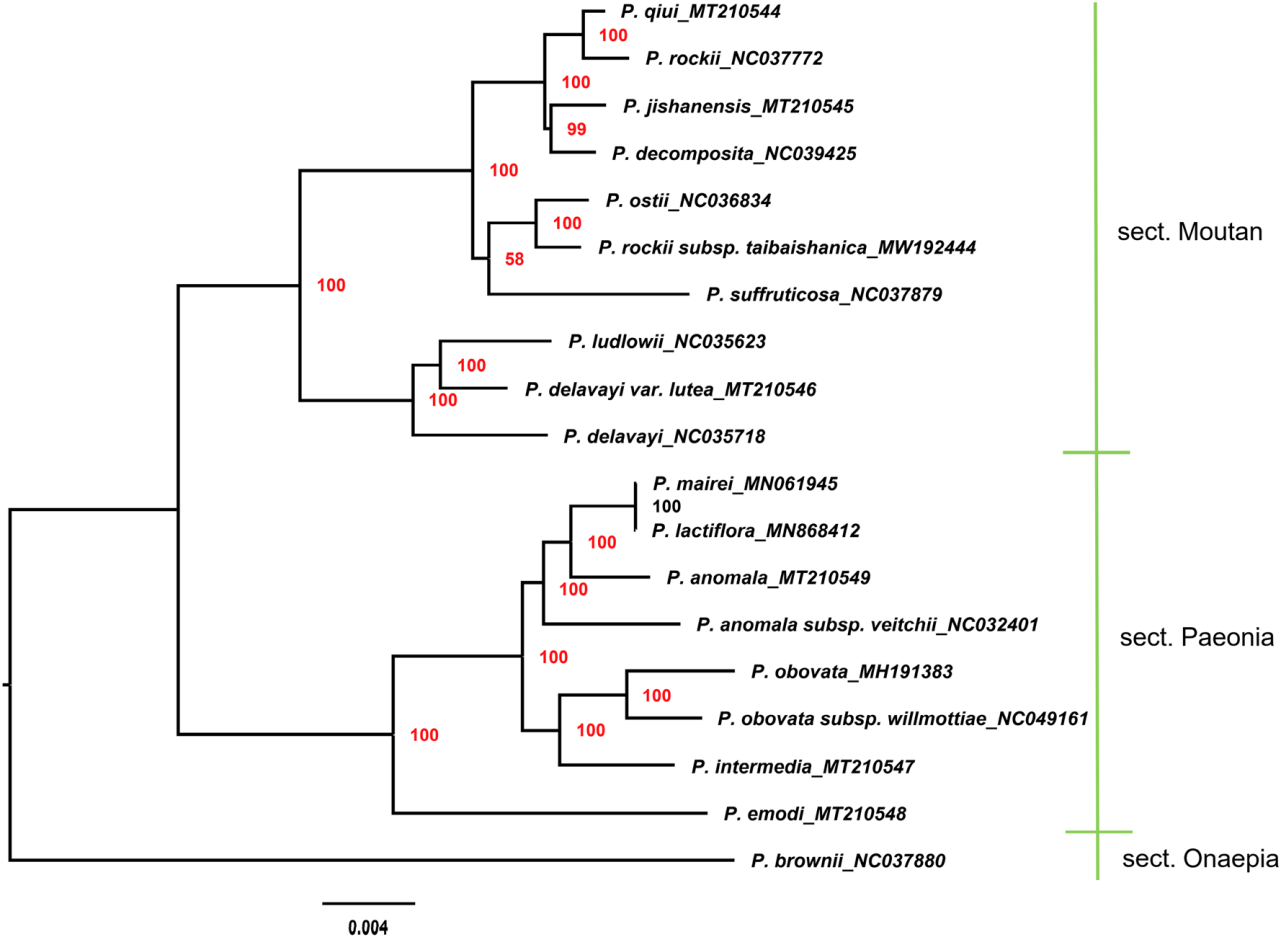
Phylogenetic tree constructed using Maximum Likelihood (ML) method based on SNPs of 19 Paeoniaceae species. Red numbers at nodes are values for bootstrap support.

Chloroplast genomes play an important role in phylogenetic studies^91,92^. In the current study, the complete chloroplast genome sequences of 19 Paeoniaceae species and 32 Ranunculaceae species were used to construct a phylogenetic tree. *Stephania tetrandra* served as the outgroup (Fig. 8). Results showed that all nodes in the phylogenetic tree had high bootstrap values. Paeoniaceae species clustered in one branch, whereas Ranunculaceae species were clearly distinguished from Paeoniaceae species, supporting the argument that Paeoniaceae is a family independent from Ranunculaceae. Species of subsect. *Vaginatae* and subsect. *Delavayanae* of sect. *Moutan* clustered in different branches, and the species relationship in sect. *Moutan* was consistent with that reported by a previous study^93^. With regard to species of sect. *Paeonia*, *P. lactiflora*, *P. anomala*, *P. anomala* subsp. *veitchii* and *P. mairei* clustered together. Pan^36^ found that *P. sterniana* is closely related to them. Xia^43^ analysed the genetic relationship of sect. *Paeonia* and found that *P. lactiflora* is closely related to *P. anomala* and *P. anomala* subsp. *veitchii*. Zhang et al.^94^ found that *P. anomala*, *P. anomala* subsp. *veitchii* and *P. mairei* are closely related and far from *P. obovata* according to the results of ML-based phylogenetic analysis using complete chloroplast genomes. The results of the current study were consistent with those of the aforementioned studies. *P. obovata*, *P. obovata* subsp. *willmottiae* and *P. intermedia* clustered together, and *P. emodi* was the sister to all other species in the sect. *Paeonia*. This branching pattern was consistent with that of the phylogenetic tree constructed by Zhou et al. by using chloroplast markers^95^. Furthermore, the relationship between sect. *Moutan* and sect. *Onaepia* was close. The result of phylogenetic tree based on SNPs (single-nucleotide polymorphisms) showed that the species relationship in Paeoniaceae was consistent with that based on the complete chloroplast genome sequences (Fig. 9). However, the relationship between sect. *Moutan* and sect. *Paeonia* was close in the tree based on SNPs, which was more coincident with the geographical distribution.

Among the 14 highly variable regions, *matK* appeared suitable for phylogenetic analysis of the species of sect. *Paeonia*, which was consistent with that based on complete chloroplast genomes. The other 13 highly variable regions were found to be unsuitable for the identification and phylogenetic analysis of Paeoniaceae species (Supplementary Fig. S1) mainly because of inadequate variations provided by a limited number of DNA loci, which was insufficient to distinguish these conservative species^96^. A previous study also demonstrated that complete chloroplast genome sequences have a higher resolution than highly variable regions and can be used to identify related species^97^, consistent with the current study.

## Materials and methods

### DNA sources

Fresh leaves of *P. intermedia*, *P. emodi* and *P. anomala* were collected from a garden in Xiaohongcun, Luanchuan County, Henan Province, China, which were transplanted from Tacheng in Xinjiang Autonomous Region (*P. intermedia* and *P. anomala*) and Shannan in Xizang Autonomous Region (*P. emodi*). These three species were identified by Prof. Peigen Xiao and Prof. Chunnian He from the Institute of Medicinal Plant Development (IMPLAD), Chinese Academy of Medical Sciences and Peking Union Medical College. Voucher specimens were deposited in the herbarium at IMPLAD, and the ID numbers were Y19075 (*P. intermedia*), Y19076 (*P. emodi*), and Y19078 (*P. anomala*). The collected fresh leaves were stored in a − 80 ℃ refrigerator until use.

### DNA extraction, sequencing, assembly and annotation

Total DNA was extracted using a DNease plant mini kit (Qiagen, Germany). Total DNA concentration was detected using a microspectrophotometer (Nanodrop 2000, USA), and total DNA quality was detected via 1% agarose gel electrophoresis. The DNA was then used to generate libraries with an average insert size of 500 bp and sequenced using Illumina Hiseq X in accordance with standard protocols. Paired-end sequencing was performed to obtain 150 bp sequences at both ends of each read. The NGS data was stated in Supplementary Table S6. Low-quality regions in the original data were trimmed by Trimmomatic software^98^, and mapped back using bwa to get the sequencing depth. The average genome coverage depth for *P. intermedia*, *P. emodi*, and *P. anomala* was 937×, 1037×, and 1113×, respectively. The Basic Local Alignment Search Tool database was constructed from the chloroplast genome sequences published on the National Centre for Biological Information. Clean reads were then compared with this database, and mapped reads were extracted according to coverage and similarity. The extracted reads were spliced into several contigs by using SOAPdenovo 2^99^ and NOVOPlasty^100^. The contigs were connected to complete chloroplast genome sequences by using the SSPACE software^101^, and gaps were filled using the GapCloser software^99^. The sequences were initially annotated by using the CPGAVAS software^102^ and the GeSeq^103^ and corrected manually. tRNAs were annotated using the tRNAscan-SE software^104^. Genes, introns and the boundaries of coding regions were compared with reference sequences.

### Structural, comparative and phylogenetic analyses

Chloroplast genome maps were generated using Chloroplot^105^ and then manually corrected. Protein functional domains of protein-coding genes were searched by Pfam^106^. The CodonW software^107^ was adopted to analyse the usage of codon. SSRs and long repeat sequences were detected using the MISA^108^ and REPuter^109^, respectively. The chloroplast genomes were compared by using the mVISTA software^84^ to detect variations within the Paeoniaceae species. Chloroplast genome sequence homology and collinearity were analysed using the Mauve software^110^. The nucleotide diversity values (Pi) of chloroplast genomes of Paeoniaceae species were computed using DnaSP v5.10^90^, and 14 highly variable regions were selected. All chloroplast genome sequences were aligned by MAFFT software^111^. Tree models were selected and phylogenetic trees were constructed by using the IQTREE software^112^. Phylogenetic trees were constructed based on the complete chloroplast genomes, SNPs, and 14 highly variable regions by ML methods. The SNPs were from the chloroplast genome sequences of Paeoniaceae species after alignment and removal of all indels^113^. ML analysis was conducted with a bootstrap of 1000 repetitions based on the TVM + F + R4 (complete chloroplast genomes), TVMe + ASC + R2 (SNPs), K3Pu + F (*accD-psaI*, *matK*, *rps3* and *rps16*), TPM2u + F (*ndhE-ndhG*), K3Pu + F + I (*ndhF*, *psbK-psbI* and *rpl16*), TIM + F + G4 (*petA-psbJ*), K2P + I (*petG-trnW-CCA*), and F81 + F (*petL-petG*, *psbH*, *rpl14-rpl16* and *rps18*) models.

All the experiment has been done in the accordance with relevant institutional, national, and international guidelines and legislation.

### Specimen collection statement

The collection of fresh leaves obtained the permission of the owner.

## Supplementary Information

Supplementary Information.

## Data Availability

The assembled chloroplast genomes of *P. intermedia*, *P. emodi* and *P. anomala* were deposited in GenBank with the accession numbers MT210547, MT210548 and MT210549. The sequences are available on NCBI now: https://www.ncbi.nlm.nih.gov/nuccore/MT210547, https://www.ncbi.nlm.nih.gov/nuccore/MT210548, https://www.ncbi.nlm.nih.gov/nuccore/MT210549.
