# Supplementary material for: Comparative and phylogenetic analyses of the chloroplast genomes of species of Paeoniaceae
Source: Sci Rep. 2021 Jul 19;11:14643. doi: 10.1038/s41598-021-94137-0 (PMC8289817; doi:10.1038/s41598-021-94137-0)
Supplement: Supplementary file 1 — Supplementary Information. [file 41598_2021_94137_MOESM1_ESM.pdf]

Comparative and Phylogenetic Analyses of the Chloroplast Genomes of 19 Species of Paeoniaceae

Liwei Wu[1,2], Liping Nie[1,2], Qing Wang[1,2], Zhichao Xu[1,2], Yu Wang[1], Chunnian He[1], Jingyuan Song[1,2] and Hui Yao[1,2,*]

[1] Key Laboratory of Bioactive Substances and Resources Utilization of Chinese Herbal Medicine, Ministry of Education, Institute of Medicinal Plant Development, Chinese Academy of Medical Sciences and Peking Union Medical College, Beijing, 100193, China;

[2] Engineering Research Center of Chinese Medicine Resources, Ministry of Education, Beijing, 100193, China

* Correspondence: scauyaoh@sina.com (H.Y.)

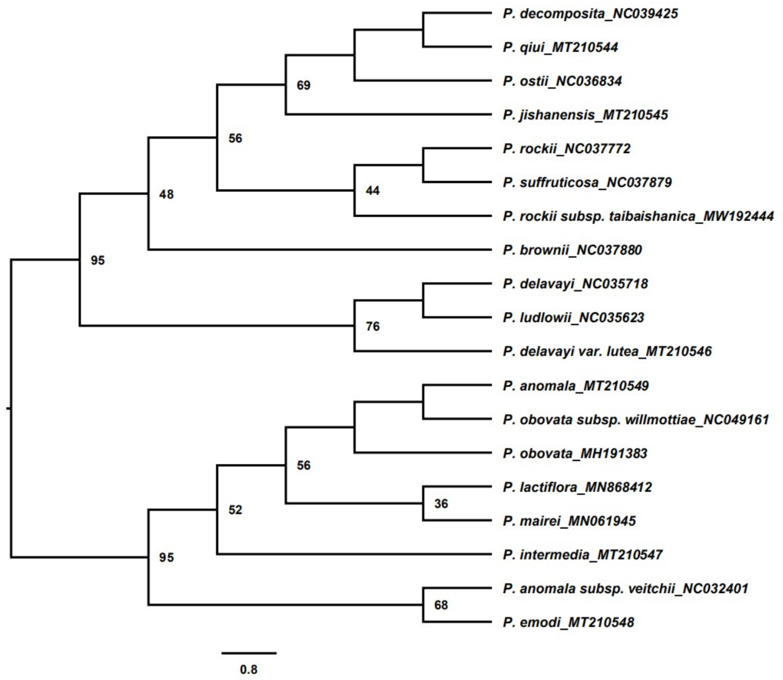

Supplementary Figure S1-1. Phylogenetic tree constructed using ML method based on *rps18* of chloroplast genomes of 19 Paeoniaceae species.

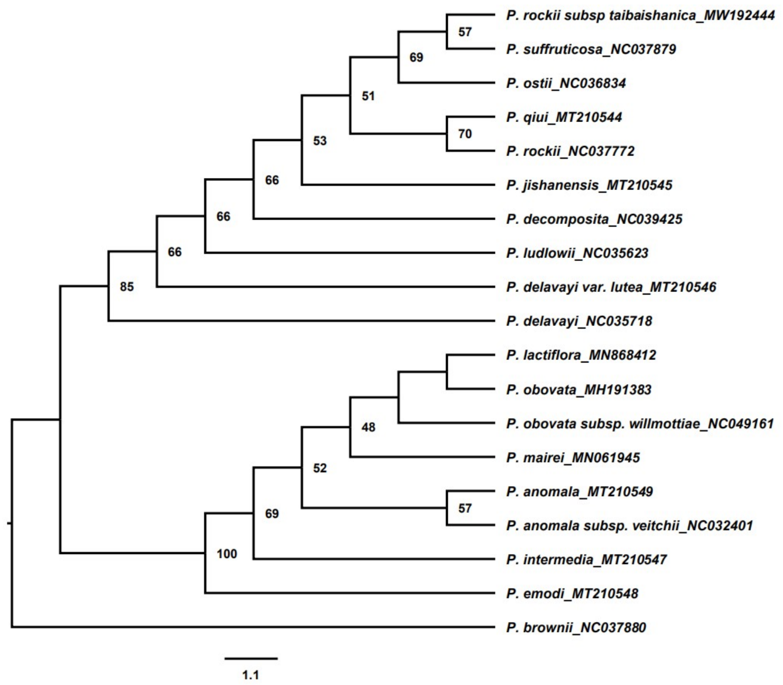

Supplementary Figure S1-2. Phylogenetic tree constructed using ML method based on *rps16* of chloroplast genomes of 19 Paeoniaceae species.

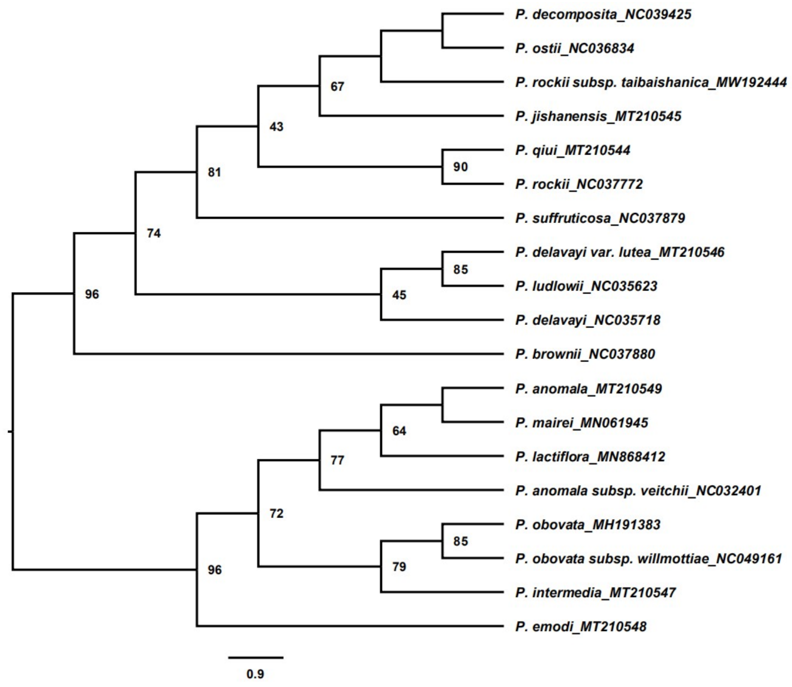

Supplementary Figure S1-3. Phylogenetic tree constructed using ML method based on *rps3* of chloroplast genomes of 19 Paeoniaceae species.

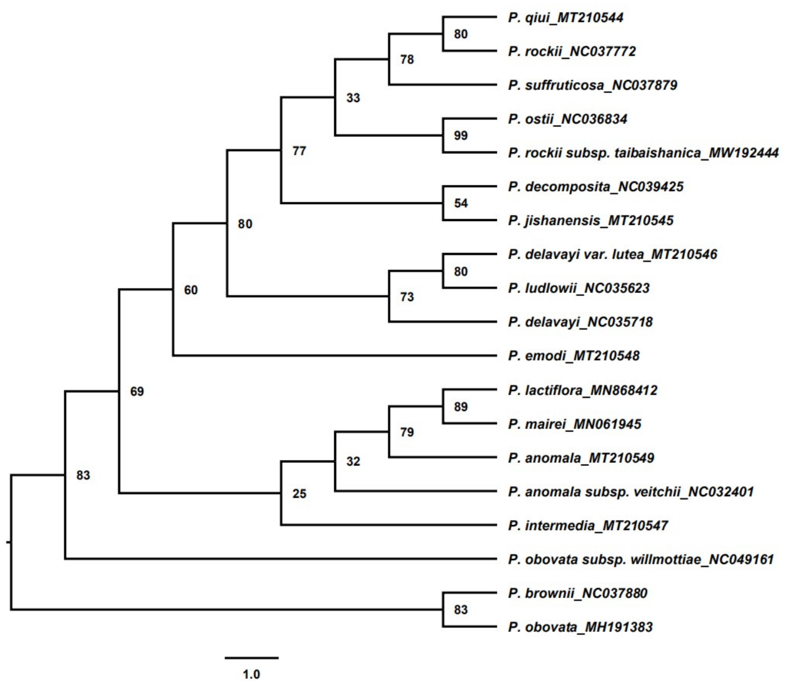

Supplementary Figure S1-4. Phylogenetic tree constructed using ML method based on *rpl16* of chloroplast genomes of 19 Paeoniaceae species.

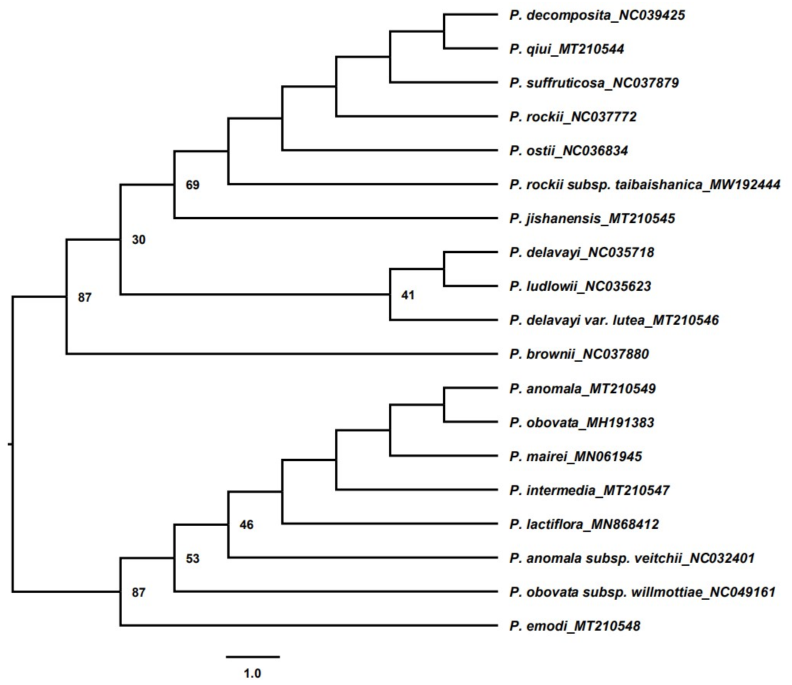

Supplementary Figure S1-5. Phylogenetic tree constructed using ML method based on *psbH* of chloroplast genomes of 19 Paeoniaceae species.

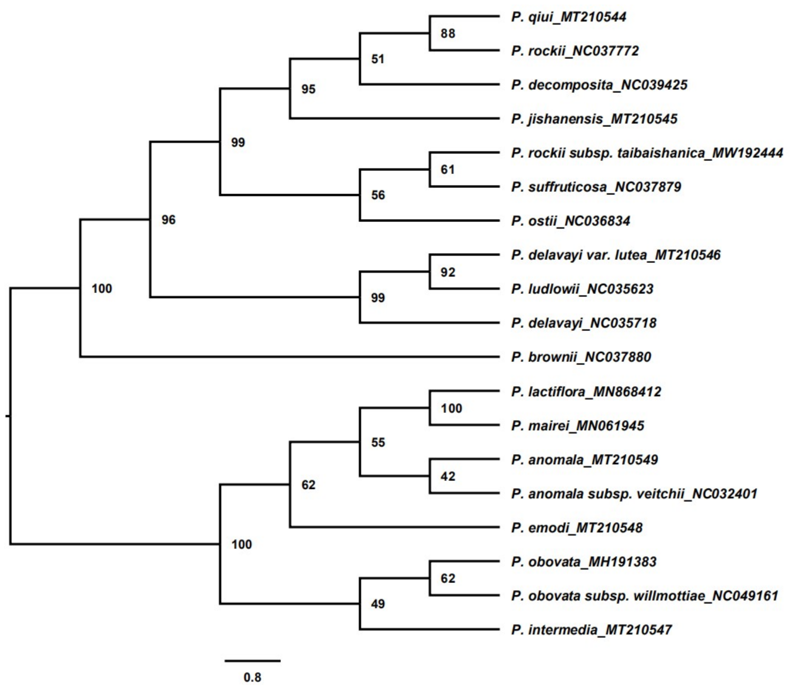

Supplementary Figure S1-6. Phylogenetic tree constructed using ML method based on *ndhF* of chloroplast genomes of 19 Paeoniaceae species.

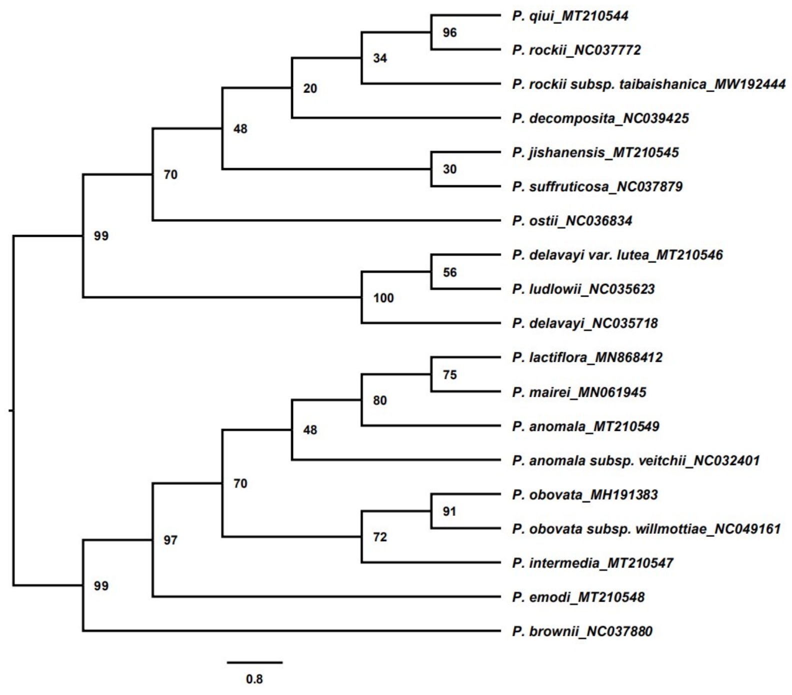

Supplementary Figure S1-7. Phylogenetic tree constructed using ML method based on *matK* of chloroplast genomes of 19 Paeoniaceae species.

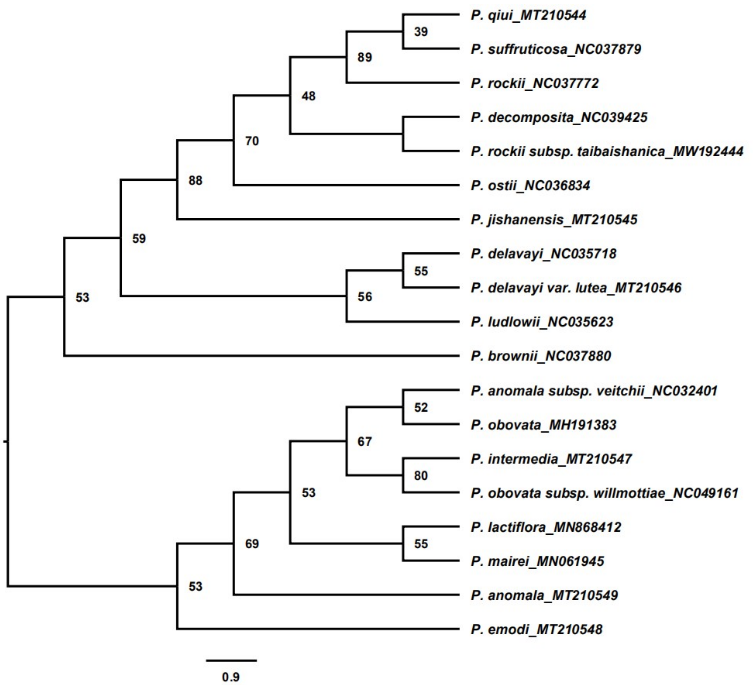

Supplementary Figure S1-8. Phylogenetic tree constructed using ML method based on *rpl14-rpl16* of chloroplast genomes of 19 Paeoniaceae species.

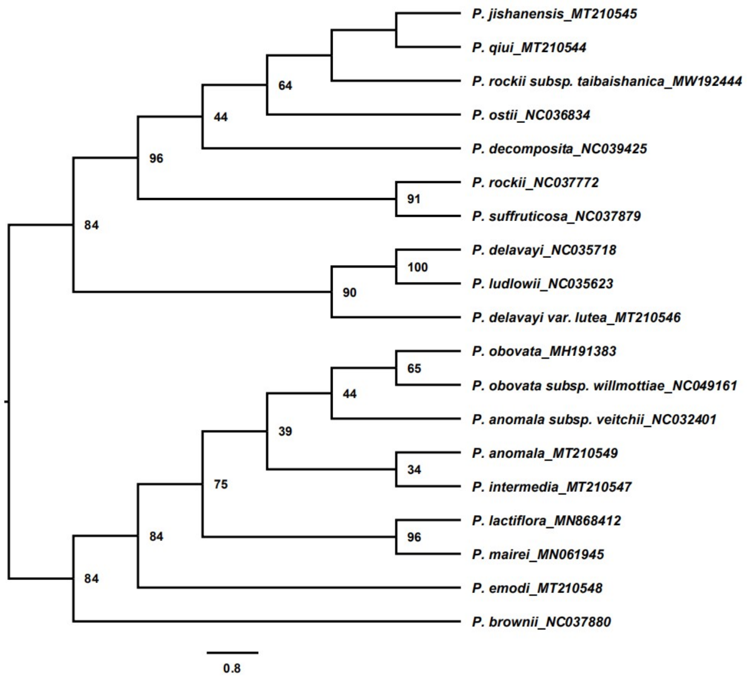

Supplementary Figure S1-9. Phylogenetic tree constructed using ML method based on *psbK-psbI* of chloroplast genomes of 19 Paeoniaceae species.

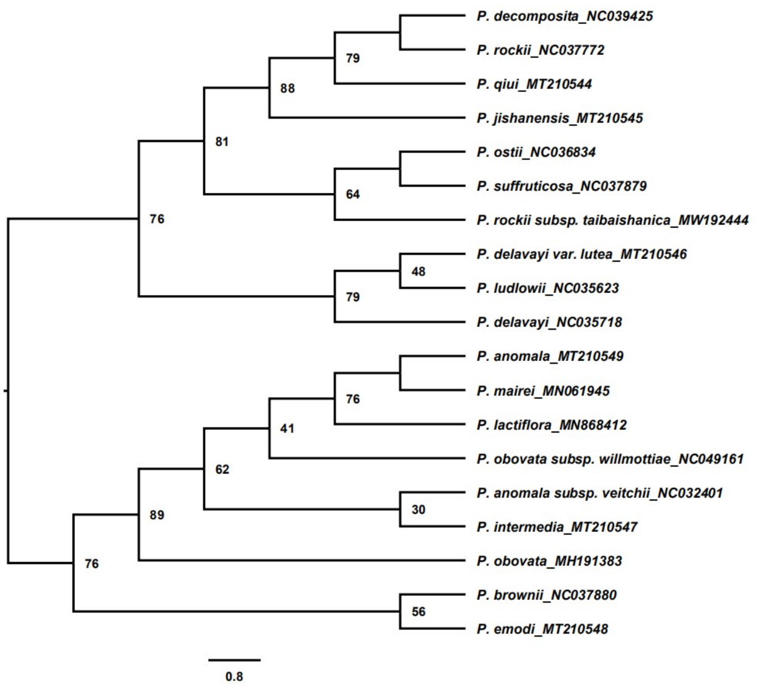

Supplementary Figure S1-10. Phylogenetic tree constructed using ML method based on *petL-petG* of chloroplast genomes of 19 Paeoniaceae species.

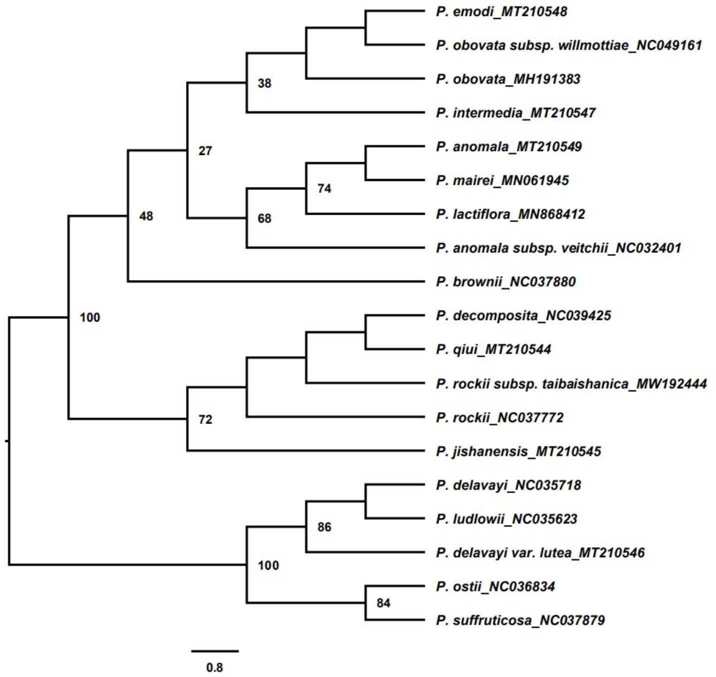

Supplementary Figure S1-11. Phylogenetic tree constructed using ML method based on *petG-trnW* of chloroplast genomes of 19 Paeoniaceae species.

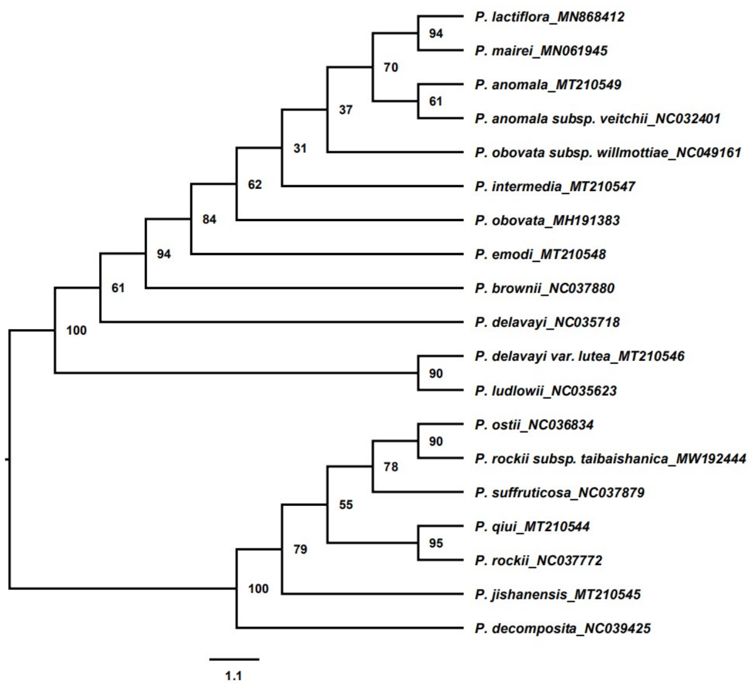

Supplementary Figure S1-12. Phylogenetic tree constructed using ML method based on *petA-psbJ* of chloroplast genomes of 19 Paeoniaceae species.

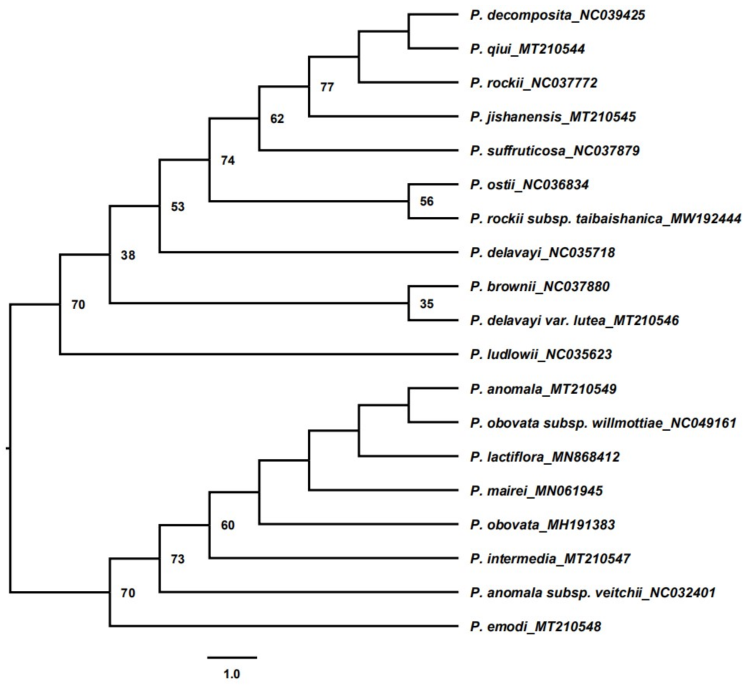

Supplementary Figure S1-13. Phylogenetic tree constructed using ML method based on *ndhE-ndhG* of chloroplast genomes of 19 Paeoniaceae species.

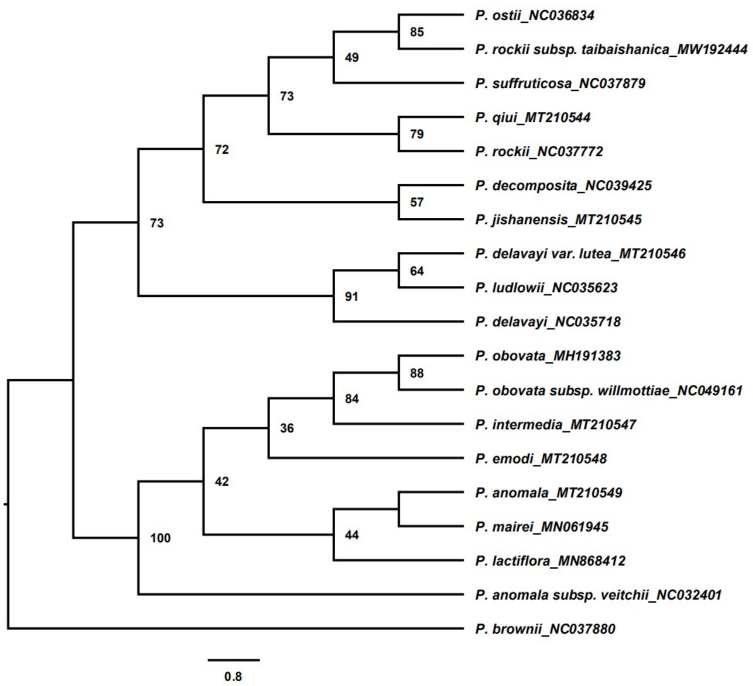

Supplementary Figure S1-14. Phylogenetic tree constructed using ML method based on *accD-psaI* of chloroplast genomes of 19 Paeoniaceae species.

Supplementary Table S1. Statistics for chloroplast genomes of the 19 Paeoniaceae species.

Latin name	Complete Genome		IRs		LSC		SSC	
	size (bp)	GC Content	size (bp)	GC Content	size (bp)	GC Content	size (bp)	GC Content
P. anomala	152,745	38.43%	25,660	43.14%	84,401	36.71%	17,025	32.77%
P. anomala subsp. *veitchii*	152,682	38.42%	25,654	43.12%	84,397	36.7%	16,978	32.72%
P. brownii	152,228	38.55%	25,645	43.16%	84,260	36.83%	16,679	33.02%
P. decomposita	152,601	38.38%	25,647	43.07%	84,272	36.69%	17,036	32.62%
P. delavayi	154,405	38.38%	25,650	43.11%	86,056	36.68%	17,050	32.73%
P. delavayi var. *lutea*	152,790	38.42%	25,649	43.1%	84,461	36.72%	17,032	32.73%
P. emodi	152,828	38.42%	25,682	43.08%	84,464	36.72%	17,001	32.82%
P. intermedia	152,713	38.43%	25,679	43.09%	84,332	36.74%	17,024	32.74%
P. jishanensis	152,631	38.35%	25,646	43.06%	84,294	36.64%	17,046	32.59%
P. lactiflora	153,405	38.43%	26,049	42.98%	84,339	36.76%	16,969	32.72%
P. ludlowii	152,687	38.44%	25,640	43.12%	84,425	36.74%	16,983	32.74%
P. mairei	152,731	38.43%	25,681	43.11%	84,401	36.73%	16,969	32.72%
P. obovata	152,736	38.43%	25,654	43.14%	84,398	36.72%	17,031	32.74%
P. obovata subsp. *willmottiae*	152,713	38.44%	25,657	43.15%	84,418	36.71%	16,982	32.8%
P. ostii	152,153	38.32%	24,864	43.09%	85,372	36.68%	17,054	32.66%
P. qiui	152,578	38.36%	25,647	43.06%	84,241	36.66%	17,044	32.62%
P. rockii	152,821	38.32%	24,729	43.08%	86,316	36.72%	17,048	32.57%
P. rockii subsp. *taibaishanica*	153,368	38.33%	25,649	43.05%	85,029	36.63%	17,042	32.61%
P. suffruticosa	153,119	38.38%	25,746	43.15%	84,569	36.64%	17,059	32.61%

Supplementary Table S2. Genes with introns in the chloroplast genomes of *P. intermedia*, *P. emodi* and *P. anomala* as well as the lengths of the exons and introns.

Gene	Location	*P. intermedia*					*P. emodi*					*P. anomala*				
		Exon I (bp)	Intron I (bp)	Exon II (bp)	Intron II (bp)	Exon III (bp)	Exon I (bp)	Intron I (bp)	Exon II (bp)	Intron II (bp)	Exon III (bp)	Exon I (bp)	Intron I (bp)	Exon II (bp)	Intron II (bp)	Exon III (bp)
atpF	LSC	159	697	414			159	698	414			159	696	414		
clpP	LSC	69	679	291	645	228	69	685	291	652	228	69	677	291	655	228
ndhA	SSC	543	1015	540			543	1016	540			543	1017	540		
ndhB	IR	777	682	756			777	682	756			777	682	756		
petB	LSC	6	752	651			6	763	651			6	764	651		
petD	LSC	9	696	474			9	694	474			9	696	474		
rpl16	LSC	9	1014	399			9	1015	399			9	1009	399		
rpl2	IR	393	668	435			393	668	435			393	668	435		
rpoC1	LSC	436	699	1616			436	702	1616			436	699	1616		
rps12	LSC	114	-	232	535	26	114	-	232	535	26	114	-	232	535	26
rps16	LSC	39	819	234			39	818	234			39	818	234		
trnA-UGC	IR	38	717	35			38	717	35			38	717	35		
trnG-UCC	LSC	34	691	48			34	691	48			34	691	48		
trnI-GAU	IR	42	933	35			42	933	35			42	933	35		
trnK-UUU	LSC	37	2452	35			37	2448	35			37	2453	35		
trnL-UAA	LSC	37	524	50			37	524	50			37	524	50		
trnV-UAC	LSC	39	573	37			39	573	37			39	573	37		
ycf3	LSC	126	723	228	753	153	126	716	228	764	153	126	727	228	749	153

Supplementary Table S3-1. Codons in chloroplast genome of *P. anomala* (MT210549).

Amino acid	Codon	No.	RSCU	Amino acid	Codon	No.	RSCU	Amino acid	Codon	No.	RSCU	Amino acid	Codon	No.	RSCU
Phe	UUU	920	1.27	Ser	UCU	541	1.64	Tyr	UAU	759	1.61	Cys	UGU	219	1.49
	UUC	534	0.73		UCC	337	1.02		UAC	183	0.39		UGC	74	0.51
Leu	UUA	830	1.85		UCA	389	1.18	TER	UAA	44	1.57	TER	UGA	20	0.71
	UUG	542	1.21		UCG	199	0.6		UAG	20	0.71	Trp	UGG	454	1
	CUU	569	1.27	Pro	CCU	407	1.47	His	CAU	478	1.55	Arg	CGU	321	1.25
	CUC	189	0.42		CCC	223	0.81		CAC	139	0.45		CGC	125	0.49
	CUA	382	0.85		CCA	299	1.08	Gln	CAA	687	1.51		CGA	336	1.31
	CUG	185	0.41		CCG	179	0.65		CAG	223	0.49		CGG	147	0.57
Ile	AUU	1074	1.46	Thr	ACU	505	1.53	Asn	AAU	957	1.55	Ser	AGU	378	1.14
	AUC	458	0.62		ACC	253	0.77		AAC	276	0.45		AGC	138	0.42
	AUA	672	0.91		ACA	401	1.22	Lys	AAA	1028	1.49	Arg	AGA	443	1.72
Met	AUG	609	1		ACG	161	0.49		AAG	350	0.51		AGG	170	0.66
Val	GUU	495	1.43	Ala	GCU	612	1.73	Asp	GAU	847	1.6	Gly	GGU	556	1.24
	GUC	163	0.47		GCC	255	0.72		GAC	215	0.4		GGC	206	0.46
	GUA	523	1.51		GCA	371	1.05	Glu	GAA	973	1.47		GGA	694	1.55
	GUG	200	0.58		GCG	176	0.5		GAG	349	0.53		GGG	336	0.75

Supplementary Table S3-2. Codons in chloroplast genome of *P. anomala* subsp. *Veitchii* (NC032401).

Amino acid	Codon	No.	RSCU	Amino acid	Codon	No.	RSCU	Amino acid	Codon	No.	RSCU	Amino acid	Codon	No.	RSCU
Phe	UUU	928	1.27	Ser	UCU	543	1.64	Tyr	UAU	757	1.61	Cys	UGU	216	1.48
	UUC	534	0.73		UCC	334	1.01		UAC	184	0.39		UGC	75	0.52
Leu	UUA	833	1.85		UCA	387	1.17	TER	UAA	43	1.54	TER	UGA	21	0.75
	UUG	543	1.2		UCG	200	0.61		UAG	20	0.71	Trp	UGG	453	1
	CUU	565	1.25	Pro	CCU	407	1.47	His	CAU	479	1.56	Arg	CGU	324	1.26
	CUC	190	0.42		CCC	220	0.79		CAC	137	0.44		CGC	126	0.49
	CUA	382	0.85		CCA	305	1.1	Gln	CAA	690	1.51		CGA	335	1.3
	CUG	192	0.43		CCG	178	0.64		CAG	223	0.49		CGG	146	0.57
Ile	AUU	1080	1.46	Thr	ACU	510	1.54	Asn	AAU	958	1.55	Ser	AGU	382	1.16
	AUC	457	0.62		ACC	252	0.76		AAC	279	0.45		AGC	136	0.41
	AUA	677	0.92		ACA	397	1.2	Lys	AAA	1034	1.49	Arg	AGA	444	1.72
Met	AUG	612	1		ACG	163	0.49		AAG	350	0.51		AGG	172	0.67
Val	GUU	497	1.44	Ala	GCU	609	1.72	Asp	GAU	848	1.6	Gly	GGU	557	1.24
	GUC	159	0.46		GCC	257	0.73		GAC	214	0.4		GGC	205	0.46
	GUA	522	1.51		GCA	372	1.05	Glu	GAA	976	1.47		GGA	694	1.55
	GUG	202	0.59		GCG	175	0.5		GAG	348	0.53		GGG	339	0.76

Supplementary Table S3-3. Codons in chloroplast genome of *P. brownii* (NC037880).

Amino acid	Codon	No.	RSCU	Amino acid	Codon	No.	RSCU	Amino acid	Codon	No.	RSCU	Amino acid	Codon	No.	RSCU
Phe	UUU	913	1.26	Ser	UCU	526	1.6	Tyr	UAU	751	1.61	Cys	UGU	216	1.49
	UUC	539	0.74		UCC	341	1.04		UAC	184	0.39		UGC	73	0.51
Leu	UUA	838	1.87		UCA	382	1.17	TER	UAA	43	1.55	TER	UGA	20	0.72
	UUG	530	1.18		UCG	205	0.63		UAG	20	0.72	Trp	UGG	454	1
	CUU	564	1.26	Pro	CCU	401	1.45	His	CAU	478	1.55	Arg	CGU	320	1.25
	CUC	193	0.43		CCC	227	0.82		CAC	140	0.45		CGC	128	0.5
	CUA	381	0.85		CCA	297	1.08	Gln	CAA	701	1.53		CGA	337	1.32
	CUG	181	0.4		CCG	179	0.65		CAG	216	0.47		CGG	145	0.57
Ile	AUU	1066	1.46	Thr	ACU	505	1.54	Asn	AAU	953	1.54	Ser	AGU	376	1.15
	AUC	456	0.62		ACC	256	0.78		AAC	284	0.46		AGC	137	0.42
	AUA	667	0.91		ACA	392	1.2	Lys	AAA	996	1.48	Arg	AGA	435	1.7
Met	AUG	605	1		ACG	157	0.48		AAG	352	0.52		AGG	166	0.65
Val	GUU	493	1.43	Ala	GCU	604	1.72	Asp	GAU	841	1.59	Gly	GGU	557	1.24
	GUC	164	0.48		GCC	254	0.72		GAC	215	0.41		GGC	206	0.46
	GUA	519	1.51		GCA	370	1.05	Glu	GAA	976	1.47		GGA	687	1.54
	GUG	199	0.58		GCG	180	0.51		GAG	352	0.53		GGG	340	0.76

Supplementary Table S3-4. Codons in chloroplast genome of *P. decomposita* (NC039425).

Amino acid	Codon	No.	RSCU	Amino acid	Codon	No.	RSCU	Amino acid	Codon	No.	RSCU	Amino acid	Codon	No.	RSCU
Phe	UUU	925	1.27	Ser	UCU	541	1.63	Tyr	UAU	766	1.61	Cys	UGU	217	1.49
	UUC	534	0.73		UCC	341	1.03		UAC	184	0.39		UGC	74	0.51
Leu	UUA	836	1.85		UCA	395	1.19	TER	UAA	42	1.5	TER	UGA	20	0.71
	UUG	539	1.19		UCG	196	0.59		UAG	22	0.79	Trp	UGG	457	1
	CUU	580	1.28	Pro	CCU	409	1.47	His	CAU	477	1.55	Arg	CGU	323	1.26
	CUC	191	0.42		CCC	223	0.8		CAC	138	0.45		CGC	123	0.48
	CUA	385	0.85		CCA	300	1.08	Gln	CAA	691	1.52		CGA	338	1.32
	CUG	185	0.41		CCG	180	0.65		CAG	218	0.48		CGG	146	0.57
Ile	AUU	1082	1.45	Thr	ACU	512	1.56	Asn	AAU	964	1.56	Ser	AGU	384	1.16
	AUC	468	0.63		ACC	247	0.75		AAC	273	0.44		AGC	136	0.41
	AUA	684	0.92		ACA	399	1.21	Lys	AAA	1020	1.5	Arg	AGA	436	1.7
Met	AUG	611	1		ACG	157	0.48		AAG	342	0.5		AGG	171	0.67
Val	GUU	491	1.43	Ala	GCU	617	1.74	Asp	GAU	854	1.6	Gly	GGU	559	1.24
	GUC	159	0.46		GCC	253	0.71		GAC	215	0.4		GGC	208	0.46
	GUA	525	1.53		GCA	372	1.05	Glu	GAA	985	1.48		GGA	692	1.54
	GUG	200	0.58		GCG	176	0.5		GAG	347	0.52		GGG	339	0.75

Supplementary Table S3-5. Codons in chloroplast genome of *P. delavayi* (NC035718).

Amino acid	Codon	No.	RSCU	Amino acid	Codon	No.	RSCU	Amino acid	Codon	No.	RSCU	Amino acid	Codon	No.	RSCU
Phe	UUU	924	1.27	Ser	UCU	542	1.63	Tyr	UAU	762	1.6	Cys	UGU	217	1.47
	UUC	532	0.73		UCC	342	1.03		UAC	190	0.4		UGC	79	0.53
Leu	UUA	838	1.84		UCA	390	1.17	TER	UAA	42	1.48	TER	UGA	20	0.71
	UUG	539	1.19		UCG	201	0.6		UAG	23	0.81	Trp	UGG	456	1
	CUU	579	1.27	Pro	CCU	404	1.44	His	CAU	477	1.55	Arg	CGU	321	1.26
	CUC	197	0.43		CCC	228	0.81		CAC	140	0.45		CGC	123	0.48
	CUA	388	0.85		CCA	308	1.1	Gln	CAA	692	1.52		CGA	337	1.32
	CUG	185	0.41		CCG	184	0.65		CAG	221	0.48		CGG	148	0.58
Ile	AUU	1077	1.45	Thr	ACU	510	1.54	Asn	AAU	960	1.55	Ser	AGU	385	1.16
	AUC	467	0.63		ACC	254	0.77		AAC	278	0.45		AGC	136	0.41
	AUA	680	0.92		ACA	400	1.21	Lys	AAA	1020	1.5	Arg	AGA	435	1.7
Met	AUG	614	1		ACG	159	0.48		AAG	344	0.5		AGG	170	0.66
Val	GUU	489	1.42	Ala	GCU	611	1.72	Asp	GAU	855	1.6	Gly	GGU	556	1.24
	GUC	163	0.47		GCC	256	0.72		GAC	217	0.4		GGC	211	0.47
	GUA	526	1.53		GCA	372	1.05	Glu	GAA	982	1.47		GGA	691	1.54
	GUG	198	0.58		GCG	182	0.51		GAG	350	0.53		GGG	341	0.76

Supplementary Table S3-6. Codons in chloroplast genome of *P. delavayi* var. *Lutea* (MT210546).

Amino acid	Codon	No.	RSCU	Amino acid	Codon	No.	RSCU	Amino acid	Codon	No.	RSCU	Amino acid	Codon	No.	RSCU
Phe	UUU	895	1.27	Ser	UCU	520	1.63	Tyr	UAU	740	1.61	Cys	UGU	213	1.47
	UUC	518	0.73		UCC	330	1.03		UAC	182	0.39		UGC	76	0.53
Leu	UUA	816	1.86		UCA	372	1.16	TER	UAA	43	1.55	TER	UGA	19	0.69
	UUG	526	1.2		UCG	191	0.6		UAG	21	0.76	Trp	UGG	446	1
	CUU	558	1.27	Pro	CCU	398	1.45	His	CAU	461	1.54	Arg	CGU	318	1.27
	CUC	185	0.42		CCC	225	0.82		CAC	137	0.46		CGC	122	0.49
	CUA	368	0.84		CCA	296	1.08	Gln	CAA	681	1.52		CGA	324	1.29
	CUG	179	0.41		CCG	177	0.65		CAG	216	0.48		CGG	145	0.58
Ile	AUU	1045	1.45	Thr	ACU	499	1.53	Asn	AAU	938	1.55	Ser	AGU	372	1.16
	AUC	454	0.63		ACC	249	0.76		AAC	271	0.45		AGC	135	0.42
	AUA	659	0.92		ACA	396	1.22	Lys	AAA	1002	1.49	Arg	AGA	433	1.72
Met	AUG	608	1		ACG	158	0.49		AAG	339	0.51		AGG	166	0.66
Val	GUU	488	1.43	Ala	GCU	601	1.72	Asp	GAU	831	1.59	Gly	GGU	549	1.24
	GUC	165	0.48		GCC	251	0.72		GAC	213	0.41		GGC	209	0.47
	GUA	515	1.51		GCA	368	1.05	Glu	GAA	964	1.48		GGA	679	1.53
	GUG	193	0.57		GCG	176	0.5		GAG	340	0.52		GGG	333	0.75

Supplementary Table S3-7. Codons in chloroplast genome of *P. emodi* (MT210548).

Amino acid	Codon	No.	RSCU	Amino acid	Codon	No.	RSCU	Amino acid	Codon	No.	RSCU	Amino acid	Codon	No.	RSCU
Phe	UUU	923	1.27	Ser	UCU	538	1.64	Tyr	UAU	759	1.61	Cys	UGU	218	1.5
	UUC	527	0.73		UCC	341	1.04		UAC	182	0.39		UGC	73	0.5
Leu	UUA	827	1.85		UCA	385	1.17	TER	UAA	43	1.57	TER	UGA	20	0.73
	UUG	538	1.2		UCG	196	0.6		UAG	19	0.7	Trp	UGG	453	1
	CUU	571	1.28	Pro	CCU	403	1.47	His	CAU	475	1.55	Arg	CGU	320	1.25
	CUC	186	0.42		CCC	221	0.81		CAC	138	0.45		CGC	127	0.5
	CUA	375	0.84		CCA	299	1.09	Gln	CAA	689	1.51		CGA	333	1.3
	CUG	184	0.41		CCG	174	0.63		CAG	222	0.49		CGG	147	0.57
Ile	AUU	1068	1.46	Thr	ACU	504	1.54	Asn	AAU	954	1.55	Ser	AGU	378	1.15
	AUC	458	0.62		ACC	249	0.76		AAC	279	0.45		AGC	135	0.41
	AUA	675	0.92		ACA	398	1.22	Lys	AAA	1017	1.49	Arg	AGA	442	1.73
Met	AUG	607	1		ACG	159	0.49		AAG	351	0.51		AGG	167	0.65
Val	GUU	493	1.44	Ala	GCU	612	1.73	Asp	GAU	836	1.59	Gly	GGU	558	1.25
	GUC	166	0.48		GCC	259	0.73		GAC	217	0.41		GGC	204	0.46
	GUA	519	1.51		GCA	367	1.04	Glu	GAA	969	1.47		GGA	688	1.54
	GUG	196	0.57		GCG	178	0.5		GAG	352	0.53		GGG	339	0.76

Supplementary Table S3-8. Codons in chloroplast genome of *P. intermedia* (MT210547).

Amino acid	Codon	No.	RSCU	Amino acid	Codon	No.	RSCU	Amino acid	Codon	No.	RSCU	Amino acid	Codon	No.	RSCU
Phe	UUU	923	1.27	Ser	UCU	540	1.64	Tyr	UAU	765	1.61	Cys	UGU	217	1.49
	UUC	533	0.73		UCC	336	1.02		UAC	183	0.39		UGC	74	0.51
Leu	UUA	830	1.85		UCA	387	1.17	TER	UAA	44	1.57	TER	UGA	20	0.71
	UUG	542	1.21		UCG	202	0.61		UAG	20	0.71	Trp	UGG	451	1
	CUU	568	1.26	Pro	CCU	407	1.47	His	CAU	481	1.56	Arg	CGU	324	1.26
	CUC	189	0.42		CCC	222	0.8		CAC	137	0.44		CGC	125	0.49
	CUA	381	0.85		CCA	302	1.09	Gln	CAA	686	1.51		CGA	334	1.3
	CUG	186	0.41		CCG	177	0.64		CAG	221	0.49		CGG	147	0.57
Ile	AUU	1076	1.47	Thr	ACU	504	1.53	Asn	AAU	956	1.55	Ser	AGU	381	1.15
	AUC	455	0.62		ACC	251	0.76		AAC	279	0.45		AGC	135	0.41
	AUA	671	0.91		ACA	403	1.22	Lys	AAA	1030	1.49	Arg	AGA	443	1.72
Met	AUG	611	1		ACG	159	0.48		AAG	349	0.51		AGG	169	0.66
Val	GUU	492	1.42	Ala	GCU	608	1.72	Asp	GAU	847	1.6	Gly	GGU	554	1.24
	GUC	165	0.48		GCC	257	0.73		GAC	215	0.4		GGC	206	0.46
	GUA	522	1.51		GCA	369	1.04	Glu	GAA	973	1.47		GGA	692	1.54
	GUG	203	0.59		GCG	179	0.51		GAG	348	0.53		GGG	342	0.76

Supplementary Table S3-9. Codons in chloroplast genome of *P. jishanensis* (MT210545).

Amino acid	Codon	No.	RSCU	Amino acid	Codon	No.	RSCU	Amino acid	Codon	No.	RSCU	Amino acid	Codon	No.	RSCU
Phe	UUU	922	1.27	Ser	UCU	538	1.64	Tyr	UAU	769	1.62	Cys	UGU	218	1.5
	UUC	532	0.73		UCC	334	1.02		UAC	181	0.38		UGC	73	0.5
Leu	UUA	836	1.86		UCA	391	1.19	TER	UAA	43	1.54	TER	UGA	20	0.71
	UUG	537	1.19		UCG	193	0.59		UAG	21	0.75	Trp	UGG	456	1
	CUU	572	1.27	Pro	CCU	407	1.47	His	CAU	475	1.54	Arg	CGU	323	1.26
	CUC	187	0.42		CCC	225	0.81		CAC	140	0.46		CGC	123	0.48
	CUA	380	0.85		CCA	301	1.09	Gln	CAA	692	1.52		CGA	338	1.31
	CUG	185	0.41		CCG	173	0.63		CAG	219	0.48		CGG	146	0.57
Ile	AUU	1071	1.46	Thr	ACU	507	1.54	Asn	AAU	964	1.56	Ser	AGU	381	1.16
	AUC	461	0.63		ACC	250	0.76		AAC	274	0.44		AGC	136	0.41
	AUA	675	0.92		ACA	398	1.21	Lys	AAA	1026	1.49	Arg	AGA	440	1.71
Met	AUG	610	1		ACG	158	0.48		AAG	347	0.51		AGG	173	0.67
Val	GUU	495	1.43	Ala	GCU	617	1.75	Asp	GAU	850	1.6	Gly	GGU	557	1.25
	GUC	161	0.47		GCC	251	0.71		GAC	214	0.4		GGC	207	0.46
	GUA	523	1.51		GCA	369	1.04	Glu	GAA	981	1.48		GGA	688	1.54
	GUG	203	0.59		GCG	176	0.5		GAG	346	0.52		GGG	336	0.75

Supplementary Table S3-10. Codons in chloroplast genome of *P. lactiflora* (MN868412).

Amino acid	Codon	No.	RSCU	Amino acid	Codon	No.	RSCU	Amino acid	Codon	No.	RSCU	Amino acid	Codon	No.	RSCU
Phe	UUU	1127	1.3	Ser	UCU	591	1.26	Tyr	UAU	891	1.38	Cys	UGU	405	0.98
	UUC	601	0.7		UCC	456	0.97		UAC	399	0.62		UGC	422	1.02
Leu	UUA	598	1.79		UCA	639	1.36	TER	UAA	480	1.07	TER	UGA	577	1.29
	UUG	385	1.15		UCG	368	0.79		UAG	287	0.64	Trp	UGG	570	1
	CUU	490	1.47	Pro	CCU	260	1.2	His	CAU	371	1.41	Arg	CGU	214	0.65
	CUC	163	0.49		CCC	185	0.85		CAC	154	0.59		CGC	132	0.4
	CUA	249	0.75		CCA	263	1.21	Gln	CAA	524	1.52		CGA	336	1.02
	CUG	120	0.36		CCG	159	0.73		CAG	165	0.48		CGG	219	0.66
Ile	AUU	930	1.53	Thr	ACU	333	1.02	Asn	AAU	920	1.39	Ser	AGU	390	0.83
	AUC	469	0.77		ACC	311	0.95		AAC	404	0.61		AGC	365	0.78
	AUA	424	0.7		ACA	418	1.28	Lys	AAA	950	1.43	Arg	AGA	683	2.07
Met	AUG	334	1		ACG	245	0.75		AAG	376	0.57		AGG	396	1.2
Val	GUU	366	1.63	Ala	GCU	277	1.28	Asp	GAU	562	1.49	Gly	GGU	329	0.85
	GUC	163	0.73		GCC	180	0.83		GAC	190	0.51		GGC	267	0.69
	GUA	237	1.06		GCA	253	1.17	Glu	GAA	719	1.47		GGA	531	1.38
	GUG	131	0.58		GCG	157	0.72		GAG	261	0.53		GGG	413	1.07

Supplementary Table S3-11. Codons in chloroplast genome of *P. ludlowii* (NC035623).

Amino acid	Codon	No.	RSCU	Amino acid	Codon	No.	RSCU	Amino acid	Codon	No.	RSCU	Amino acid	Codon	No.	RSCU
Phe	UUU	928	1.27	Ser	UCU	549	1.64	Tyr	UAU	768	1.6	Cys	UGU	218	1.47
	UUC	536	0.73		UCC	347	1.04		UAC	191	0.4		UGC	79	0.53
Leu	UUA	844	1.85		UCA	392	1.17	TER	UAA	44	1.52	TER	UGA	20	0.69
	UUG	540	1.18		UCG	199	0.59		UAG	23	0.79	Trp	UGG	458	1
	CUU	585	1.28	Pro	CCU	408	1.44	His	CAU	478	1.54	Arg	CGU	324	1.25
	CUC	197	0.43		CCC	229	0.81		CAC	141	0.46		CGC	126	0.49
	CUA	393	0.86		CCA	310	1.09	Gln	CAA	698	1.51		CGA	339	1.31
	CUG	185	0.4		CCG	188	0.66		CAG	224	0.49		CGG	151	0.58
Ile	AUU	1082	1.45	Thr	ACU	515	1.55	Asn	AAU	969	1.55	Ser	AGU	384	1.15
	AUC	470	0.63		ACC	255	0.77		AAC	283	0.45		AGC	139	0.41
	AUA	683	0.92		ACA	400	1.2	Lys	AAA	1029	1.49	Arg	AGA	440	1.7
Met	AUG	617	1		ACG	163	0.49		AAG	348	0.51		AGG	170	0.66
Val	GUU	494	1.42	Ala	GCU	617	1.73	Asp	GAU	856	1.59	Gly	GGU	561	1.24
	GUC	166	0.48		GCC	259	0.73		GAC	220	0.41		GGC	212	0.47
	GUA	529	1.52		GCA	373	1.05	Glu	GAA	986	1.48		GGA	695	1.53
	GUG	202	0.58		GCG	178	0.5		GAG	350	0.52		GGG	347	0.76

Supplementary Table S3-12. Codons in chloroplast genome of *P. mairei* (MN061945).

Amino acid	Codon	No.	RSCU	Amino acid	Codon	No.	RSCU	Amino acid	Codon	No.	RSCU	Amino acid	Codon	No.	RSCU
Phe	UUU	930	1.27	Ser	UCU	546	1.63	Tyr	UAU	769	1.6	Cys	UGU	219	1.48
	UUC	537	0.73		UCC	345	1.03		UAC	191	0.4		UGC	77	0.52
Leu	UUA	836	1.83		UCA	397	1.18	TER	UAA	45	1.55	TER	UGA	20	0.69
	UUG	547	1.2		UCG	202	0.6		UAG	22	0.76	Trp	UGG	456	1
	CUU	581	1.27	Pro	CCU	413	1.46	His	CAU	480	1.55	Arg	CGU	324	1.25
	CUC	196	0.43		CCC	223	0.79		CAC	138	0.45		CGC	125	0.48
	CUA	392	0.86		CCA	310	1.1	Gln	CAA	692	1.51		CGA	342	1.32
	CUG	184	0.4		CCG	186	0.66		CAG	227	0.49		CGG	148	0.57
Ile	AUU	1091	1.46	Thr	ACU	514	1.54	Asn	AAU	968	1.55	Ser	AGU	383	1.14
	AUC	466	0.62		ACC	258	0.77		AAC	280	0.45		AGC	139	0.41
	AUA	680	0.91		ACA	404	1.21	Lys	AAA	1028	1.49	Arg	AGA	445	1.72
Met	AUG	612	1		ACG	159	0.48		AAG	349	0.51		AGG	170	0.66
Val	GUU	493	1.41	Ala	GCU	616	1.72	Asp	GAU	856	1.6	Gly	GGU	564	1.24
	GUC	168	0.48		GCC	261	0.73		GAC	216	0.4		GGC	206	0.45
	GUA	531	1.52		GCA	374	1.05	Glu	GAA	978	1.47		GGA	705	1.55
	GUG	202	0.58		GCG	178	0.5		GAG	354	0.53		GGG	343	0.75

Supplementary Table S3-13. Codons in chloroplast genome of *P. obovata* (MH191383).

Amino acid	Codon	No.	RSCU	Amino acid	Codon	No.	RSCU	Amino acid	Codon	No.	RSCU	Amino acid	Codon	No.	RSCU
Phe	UUU	1113	1.29	Ser	UCU	582	1.24	Tyr	UAU	893	1.39	Cys	UGU	417	1.01
	UUC	613	0.71		UCC	443	0.95		UAC	389	0.61		UGC	411	0.99
Leu	UUA	601	1.75		UCA	627	1.34	TER	UAA	452	1.11	TER	UGA	509	1.25
	UUG	402	1.17		UCG	370	0.79		UAG	257	0.63	Trp	UGG	588	1
	CUU	489	1.43	Pro	CCU	248	1.14	His	CAU	378	1.42	Arg	CGU	206	0.63
	CUC	170	0.5		CCC	195	0.89		CAC	153	0.58		CGC	141	0.43
	CUA	265	0.77		CCA	261	1.2	Gln	CAA	496	1.49		CGA	323	0.99
	CUG	128	0.37		CCG	168	0.77		CAG	170	0.51		CGG	229	0.7
Ile	AUU	912	1.51	Thr	ACU	336	1.03	Asn	AAU	977	1.41	Ser	AGU	413	0.88
	AUC	454	0.75		ACC	298	0.92		AAC	407	0.59		AGC	377	0.8
	AUA	445	0.74		ACA	425	1.31	Lys	AAA	942	1.4	Arg	AGA	647	1.99
Met	AUG	345	1		ACG	242	0.74		AAG	405	0.6		AGG	408	1.25
Val	GUU	351	1.66	Ala	GCU	265	1.28	Asp	GAU	599	1.49	Gly	GGU	316	0.82
	GUC	148	0.7		GCC	168	0.81		GAC	206	0.51		GGC	268	0.7
	GUA	217	1.03		GCA	247	1.19	Glu	GAA	679	1.44		GGA	536	1.4
	GUG	129	0.61		GCG	149	0.72		GAG	262	0.56		GGG	415	1.08

Supplementary Table S3-14. Codons in chloroplast genome of *P. obovata* subsp. *Willmottiae* (NC049161).

Amino acid	Codon	No.	RSCU	Amino acid	Codon	No.	RSCU	Amino acid	Codon	No.	RSCU	Amino acid	Codon	No.	RSCU
Phe	UUU	924	1.27	Ser	UCU	541	1.63	Tyr	UAU	764	1.61	Cys	UGU	217	1.5
	UUC	535	0.73		UCC	337	1.02		UAC	185	0.39		UGC	73	0.5
Leu	UUA	828	1.84		UCA	387	1.17	TER	UAA	43	1.54	TER	UGA	21	0.75
	UUG	542	1.2		UCG	202	0.61		UAG	20	0.71	Trp	UGG	453	1
	CUU	570	1.27	Pro	CCU	406	1.46	His	CAU	480	1.56	Arg	CGU	322	1.26
	CUC	191	0.42		CCC	227	0.82		CAC	137	0.44		CGC	126	0.49
	CUA	383	0.85		CCA	300	1.08	Gln	CAA	689	1.51		CGA	332	1.3
	CUG	185	0.41		CCG	180	0.65		CAG	224	0.49		CGG	150	0.59
Ile	AUU	1072	1.46	Thr	ACU	505	1.54	Asn	AAU	961	1.56	Ser	AGU	382	1.15
	AUC	457	0.62		ACC	249	0.76		AAC	275	0.44		AGC	138	0.42
	AUA	672	0.92		ACA	402	1.22	Lys	AAA	1023	1.49	Arg	AGA	438	1.71
Met	AUG	611	1		ACG	159	0.48		AAG	348	0.51		AGG	170	0.66
Val	GUU	494	1.44	Ala	GCU	608	1.72	Asp	GAU	849	1.6	Gly	GGU	556	1.24
	GUC	163	0.47		GCC	255	0.72		GAC	215	0.4		GGC	205	0.46
	GUA	522	1.52		GCA	371	1.05	Glu	GAA	969	1.47		GGA	693	1.54
	GUG	198	0.58		GCG	179	0.51		GAG	346	0.53		GGG	344	0.77

Supplementary Table S3-15. Codons in chloroplast genome of *P. ostii* (NC036834).

Amino acid	Codon	No.	RSCU	Amino acid	Codon	No.	RSCU	Amino acid	Codon	No.	RSCU	Amino acid	Codon	No.	RSCU
Phe	UUU	929	1.26	Ser	UCU	543	1.64	Tyr	UAU	766	1.62	Cys	UGU	218	1.49
	UUC	541	0.74		UCC	340	1.03		UAC	179	0.38		UGC	74	0.51
Leu	UUA	831	1.85		UCA	390	1.18	TER	UAA	44	1.57	TER	UGA	20	0.71
	UUG	530	1.18		UCG	195	0.59		UAG	20	0.71	Trp	UGG	452	1
	CUU	577	1.29	Pro	CCU	406	1.47	His	CAU	473	1.53	Arg	CGU	314	1.23
	CUC	195	0.44		CCC	222	0.8		CAC	144	0.47		CGC	126	0.49
	CUA	380	0.85		CCA	298	1.08	Gln	CAA	694	1.52		CGA	343	1.35
	CUG	176	0.39		CCG	180	0.65		CAG	219	0.48		CGG	143	0.56
Ile	AUU	1072	1.46	Thr	ACU	507	1.54	Asn	AAU	967	1.55	Ser	AGU	382	1.15
	AUC	464	0.63		ACC	252	0.77		AAC	277	0.45		AGC	137	0.41
	AUA	672	0.91		ACA	398	1.21	Lys	AAA	1011	1.49	Arg	AGA	433	1.7
Met	AUG	619	1		ACG	157	0.48		AAG	349	0.51		AGG	171	0.67
Val	GUU	498	1.44	Ala	GCU	613	1.74	Asp	GAU	854	1.6	Gly	GGU	548	1.23
	GUC	163	0.47		GCC	252	0.72		GAC	211	0.4		GGC	208	0.47
	GUA	519	1.5		GCA	368	1.05	Glu	GAA	994	1.49		GGA	680	1.53
	GUG	203	0.59		GCG	173	0.49		GAG	343	0.51		GGG	340	0.77

Supplementary Table S3-16. Codons in chloroplast genome of *P. qiui* (MT210544).

Amino acid	Codon	No.	RSCU	Amino acid	Codon	No.	RSCU	Amino acid	Codon	No.	RSCU	Amino acid	Codon	No.	RSCU
Phe	UUU	923	1.27	Ser	UCU	538	1.64	Tyr	UAU	767	1.62	Cys	UGU	217	1.49
	UUC	532	0.73		UCC	335	1.02		UAC	181	0.38		UGC	74	0.51
Leu	UUA	837	1.86		UCA	391	1.19	TER	UAA	43	1.54	TER	UGA	20	0.71
	UUG	536	1.19		UCG	193	0.59		UAG	21	0.75	Trp	UGG	455	1
	CUU	572	1.27	Pro	CCU	408	1.47	His	CAU	476	1.55	Arg	CGU	324	1.26
	CUC	187	0.42		CCC	223	0.81		CAC	140	0.45		CGC	123	0.48
	CUA	377	0.84		CCA	301	1.09	Gln	CAA	692	1.52		CGA	339	1.32
	CUG	184	0.41		CCG	176	0.64		CAG	219	0.48		CGG	146	0.57
Ile	AUU	1071	1.46	Thr	ACU	506	1.54	Asn	AAU	963	1.56	Ser	AGU	381	1.16
	AUC	460	0.63		ACC	250	0.76		AAC	275	0.44		AGC	135	0.41
	AUA	675	0.92		ACA	398	1.21	Lys	AAA	1028	1.5	Arg	AGA	440	1.71
Met	AUG	612	1		ACG	157	0.48		AAG	347	0.5		AGG	173	0.67
Val	GUU	499	1.44	Ala	GCU	618	1.75	Asp	GAU	849	1.6	Gly	GGU	554	1.24
	GUC	160	0.46		GCC	251	0.71		GAC	215	0.4		GGC	208	0.47
	GUA	522	1.51		GCA	368	1.04	Glu	GAA	980	1.48		GGA	689	1.54
	GUG	202	0.58		GCG	176	0.5		GAG	346	0.52		GGG	337	0.75

Supplementary Table S3-17. Codons in chloroplast genome of *P. rockii* (NC037772).

Amino acid	Codon	No.	RSCU	Amino acid	Codon	No.	RSCU	Amino acid	Codon	No.	RSCU	Amino acid	Codon	No.	RSCU
Phe	UUU	1111	1.29	Ser	UCU	588	1.25	Tyr	UAU	904	1.4	Cys	UGU	420	1.01
	UUC	608	0.71		UCC	442	0.94		UAC	392	0.6		UGC	409	0.99
Leu	UUA	601	1.75		UCA	629	1.34	TER	UAA	451	1.11	TER	UGA	509	1.26
	UUG	406	1.18		UCG	368	0.78		UAG	256	0.63	Trp	UGG	596	1
	CUU	491	1.43	Pro	CCU	251	1.14	His	CAU	376	1.43	Arg	CGU	199	0.61
	CUC	164	0.48		CCC	199	0.9		CAC	151	0.57		CGC	143	0.44
	CUA	265	0.77		CCA	265	1.2	Gln	CAA	496	1.49		CGA	328	1.01
	CUG	129	0.38		CCG	169	0.76		CAG	170	0.51		CGG	221	0.68
Ile	AUU	915	1.51	Thr	ACU	338	1.04	Asn	AAU	985	1.42	Ser	AGU	414	0.88
	AUC	458	0.76		ACC	299	0.92		AAC	403	0.58		AGC	381	0.81
	AUA	446	0.74		ACA	422	1.3	Lys	AAA	938	1.39	Arg	AGA	649	2
Met	AUG	351	1		ACG	244	0.75		AAG	409	0.61		AGG	411	1.26
Val	GUU	350	1.66	Ala	GCU	264	1.29	Asp	GAU	599	1.49	Gly	GGU	313	0.82
	GUC	146	0.69		GCC	164	0.8		GAC	203	0.51		GGC	268	0.7
	GUA	221	1.05		GCA	245	1.2	Glu	GAA	686	1.45		GGA	530	1.39
	GUG	128	0.61		GCG	144	0.71		GAG	258	0.55		GGG	412	1.08

Supplementary Table S3-18. Codons in chloroplast genome of *P. rockii* subsp. *Taibaishanica* (MW192444).

Amino acid	Codon	No.	RSCU	Amino acid	Codon	No.	RSCU	Amino acid	Codon	No.	RSCU	Amino acid	Codon	No.	RSCU
Phe	UUU	1111	1.29	Ser	UCU	588	1.25	Tyr	UAU	908	1.4	Cys	UGU	419	1.01
	UUC	609	0.71		UCC	443	0.94		UAC	392	0.6		UGC	408	0.99
Leu	UUA	601	1.75		UCA	630	1.34	TER	UAA	450	1.11	TER	UGA	508	1.25
	UUG	403	1.18		UCG	363	0.77		UAG	259	0.64	Trp	UGG	597	1
	CUU	492	1.44	Pro	CCU	251	1.13	His	CAU	373	1.42	Arg	CGU	199	0.61
	CUC	165	0.48		CCC	198	0.89		CAC	152	0.58		CGC	142	0.44
	CUA	266	0.78		CCA	269	1.21	Gln	CAA	494	1.49		CGA	329	1.01
	CUG	129	0.38		CCG	169	0.76		CAG	169	0.51		CGG	222	0.68
Ile	AUU	914	1.51	Thr	ACU	337	1.04	Asn	AAU	982	1.42	Ser	AGU	413	0.88
	AUC	458	0.76		ACC	299	0.92		AAC	405	0.58		AGC	379	0.81
	AUA	447	0.74		ACA	415	1.28	Lys	AAA	941	1.4	Arg	AGA	649	1.99
Met	AUG	349	1		ACG	245	0.76		AAG	407	0.6		AGG	411	1.26
Val	GUU	352	1.66	Ala	GCU	265	1.29	Asp	GAU	599	1.49	Gly	GGU	314	0.82
	GUC	146	0.69		GCC	165	0.8		GAC	203	0.51		GGC	270	0.71
	GUA	221	1.04		GCA	248	1.21	Glu	GAA	685	1.45		GGA	531	1.39
	GUG	129	0.61		GCG	145	0.7		GAG	258	0.55		GGG	411	1.08

Supplementary Table S3-19. Codons in chloroplast genome of *P. suffruticosa* (NC037879).

Amino acid	Codon	No.	RSCU	Amino acid	Codon	No.	RSCU	Amino acid	Codon	No.	RSCU	Amino acid	Codon	No.	RSCU
Phe	UUU	912	1.26	Ser	UCU	531	1.63	Tyr	UAU	763	1.61	Cys	UGU	214	1.49
	UUC	535	0.74		UCC	338	1.04		UAC	183	0.39		UGC	73	0.51
Leu	UUA	833	1.86		UCA	388	1.19	TER	UAA	42	1.52	TER	UGA	20	0.72
	UUG	534	1.2		UCG	194	0.59		UAG	21	0.76	Trp	UGG	450	1
	CUU	568	1.27	Pro	CCU	402	1.46	His	CAU	475	1.55	Arg	CGU	319	1.25
	CUC	184	0.41		CCC	225	0.82		CAC	139	0.45		CGC	120	0.47
	CUA	377	0.84		CCA	300	1.09	Gln	CAA	689	1.52		CGA	335	1.31
	CUG	185	0.41		CCG	175	0.64		CAG	219	0.48		CGG	144	0.57
Ile	AUU	1067	1.46	Thr	ACU	503	1.54	Asn	AAU	963	1.56	Ser	AGU	374	1.15
	AUC	460	0.63		ACC	248	0.76		AAC	273	0.44		AGC	134	0.41
	AUA	672	0.92		ACA	395	1.21	Lys	AAA	1016	1.49	Arg	AGA	440	1.73
Met	AUG	608	1		ACG	159	0.49		AAG	347	0.51		AGG	171	0.67
Val	GUU	492	1.44	Ala	GCU	606	1.73	Asp	GAU	846	1.6	Gly	GGU	555	1.25
	GUC	161	0.47		GCC	251	0.72		GAC	212	0.4		GGC	206	0.46
	GUA	515	1.5		GCA	366	1.05	Glu	GAA	971	1.48		GGA	681	1.53
	GUG	203	0.59		GCG	176	0.5		GAG	340	0.52		GGG	336	0.76

Supplementary Table S4. Repeat sequences in chloroplast genomes of 19 Paeoniaceae species.

Species	Complement	Forward					Palindrome					Reverse
	30-39	30-39	40-49	50-59	60-69	≥70	30-39	40-49	50-59	60-69	≥70	30-39
P. anomala	5	16	3	2	2		15	4	2	2		4
P. anomala subsp. *veitchii*	5	16	3	2	2		15	4	2	2		4
P. brownii		13	7		3	4	12	5		4	4	
P. decomposita		13	4	4			13	6	4			
P. delavayi		12	4	4			12	6	4			1
P. delavayi var. *lutea*		14	4	4			13	5	4			
P. emodi		14	4	4			10	5	4			
P. intermedia		14	3	2	2		14	4	2	2		
P. jishanensis		14	4	4			12	6	4			
P. lactiflora	2	14	5	2	2		13	5	2	2		1
P. ludlowii		12	3	2	2		13	3	2	2		1
P. mairei	2	14	5	2	2		13	5	2	2		1
P. obovata		12	3	2	2		12	3	2	2		1
P. obovata subsp. *willmottiae*		10	3	2	2		11	3	2	2		1
P. ostii		14	4	4			12	6	4		1	
P. qiui		13	4	4			12	6	4			1
P. rockii	1	19	5	5			20	6	5			2
P. rockii subsp. *taibaishanica*		13	5	4		1	13	6	4		1	
P. suffruticosa		17	8	4			11	10	4			

Supplementary Table S5. SSRs in the chloroplast genomes of 19 Paeoniaceae species.

Species	A/T	C/G	AG/CT	AT/AT	AAT/ATT	ACG/CGT	AAAC/ GTTT	AAAG/ CTTT	AAAT/ ATTT	AGAT/ ATCT	AAAAG/ CTTTT	AATAT/ ATATT	total
P. anomala	44	1	1	12	6	0	1	0	2	1	1	0	69
P. anomala subsp. *veitchii*	44	1	1	11	7	0	1	0	2	1	0	0	68
P. brownii	43	2	1	8	7	0	1	0	3	1	0	0	66
P. decomposita	42	2	1	11	7	0	1	1	2	1	0	0	68
P. delavayi	40	1	1	11	5	1	1	0	2	1	0	0	63
P. delavayi var. *lutea*	39	0	1	12	5	0	1	0	2	1	0	0	61
P. emodi	47	2	1	13	7	0	1	0	2	1	0	0	74
P. intermedia	44	2	1	12	6	0	1	0	2	1	0	0	69
P. jishanensis	46	1	1	12	6	0	1	1	2	1	0	1	72
P. lactiflora	42	2	1	12	7	0	1	0	2	1	0	0	68
P. ludlowii	42	1	1	11	5	0	1	0	2	1	0	0	64
P. mairei	42	2	1	12	7	0	1	0	2	1	0	0	68
P. obovata	42	1	1	13	7	0	1	0	2	1	0	0	68
P. obovata subsp. *willmottiae*	44	2	1	13	7	0	1	0	2	1	0	0	71
P. ostii	41	1	1	13	6	0	1	2	2	1	0	0	68
P. qiui	46	3	1	11	7	0	1	1	2	1	0	0	73
P. rockii	46	3	1	12	7	0	1	1	2	1	0	0	74
P. rockii subsp. *taibaishanica*	40	1	1	12	6	0	1	2	2	1	0	0	66
P. suffruticosa	44	4	1	11	7	0	1	1	2	1	0	0	72

Supplementary Table S6. Statement of the NGS data.

Raw data					
Sample ID	Total Reads	Total Bases	Q20%	Q30%	GC%
Y19075	57,254,022	8,588,103,300	96.48	90.38	35.32
Y19076	42,785,582	6,417,837,300	96.38	90.26	36.04
Y19078	52,484,402	7,872,660,300	96.18	89.83	35.98
Clean data					
Sample ID	Total Reads	Total Bases	Q20%	Q30%	GC%
Y19075	51,533,194	7,636,952,209	97.88	92.53	35.05
Y19076	38,027,786	5,635,808,706	97.88	92.55	35.76
Y19078	46,457,850	6,875,374,599	97.74	92.2	35.71
